# cgDist: Nucleotide-level distance calculation from cgMLST allelic profiles

**DOI:** 10.1093/nargab/lqag090

**Published:** 2026-09-02

**Authors:** Andrea de Ruvo, Pierluigi Castelli, Andrea Bucciacchio, Iolanda Mangone, Verónica Mixão, Vítor Borges, Michele Flammini, Nicolas Radomski, Adriano Di Pasquale

**Affiliations:** Computer Science, Gran Sasso Science Institute, 67100 L’Aquila, Italy; National Reference Centre (NRC) for Whole Genome Sequencing of microbial pathogens: database and bioinformatics analysis (GENPAT), Istituto Zooprofilattico Sperimentale dell’Abruzzo e del Molise “G. Caporale”, 64100 Teramo, Italy; National Reference Centre (NRC) for Whole Genome Sequencing of microbial pathogens: database and bioinformatics analysis (GENPAT), Istituto Zooprofilattico Sperimentale dell’Abruzzo e del Molise “G. Caporale”, 64100 Teramo, Italy; National Reference Centre (NRC) for Whole Genome Sequencing of microbial pathogens: database and bioinformatics analysis (GENPAT), Istituto Zooprofilattico Sperimentale dell’Abruzzo e del Molise “G. Caporale”, 64100 Teramo, Italy; National Reference Centre (NRC) for Whole Genome Sequencing of microbial pathogens: database and bioinformatics analysis (GENPAT), Istituto Zooprofilattico Sperimentale dell’Abruzzo e del Molise “G. Caporale”, 64100 Teramo, Italy; Genomics and Bioinformatics Unit, Department of Infectious Diseases, Instituto Nacional de Saúde Doutor Ricardo Jorge, 1649-016 Lisbon, Portugal; Genomics and Bioinformatics Unit, Department of Infectious Diseases, Instituto Nacional de Saúde Doutor Ricardo Jorge, 1649-016 Lisbon, Portugal; Computer Science, Gran Sasso Science Institute, 67100 L’Aquila, Italy; National Reference Centre (NRC) for Whole Genome Sequencing of microbial pathogens: database and bioinformatics analysis (GENPAT), Istituto Zooprofilattico Sperimentale dell’Abruzzo e del Molise “G. Caporale”, 64100 Teramo, Italy; National Reference Centre (NRC) for Whole Genome Sequencing of microbial pathogens: database and bioinformatics analysis (GENPAT), Istituto Zooprofilattico Sperimentale dell’Abruzzo e del Molise “G. Caporale”, 64100 Teramo, Italy

## Abstract

Bacterial genomic surveillance requires balancing computational efficiency with genetic resolution for effective cluster investigation. cgMLST distance calculations treat all allelic differences as equivalent units, obscuring nucleotide-level variation. Furthermore, single nucleotide polymorphism-based pipelines provide finer resolution at substantially higher computational cost, which limits their routine deployment in surveillance laboratories. We present cgDist, an algorithm that calculates nucleotide-level distances directly from cgMLST allelic profiles, providing finer resolution than allele-count distances by leveraging within-allele nucleotide variation. The cache architecture stores alignment statistics, enabling distance calculation modes without computation and supporting both dataset-specific and schema-complete cache generation. This design enables incremental surveillance analysis, with performance benefits as laboratories accumulate alignment data. cgDist functions as a precision ‘zoom lens’ for the investigation of clusters identified through initial cgMLST screening. Rather than restructuring population relationships, this targeted approach concentrates enhanced resolution where it is most informative. The algorithm ensures that cgDist distances are greater than or equal to corresponding cgMLST distances, preserving epidemiological interpretability while adding genetic discrimination. By increasing resolution within identified clusters, cgDist may also support outbreak investigation, a potential application that remains to be evaluated on outbreak-derived data.

## Graphical Abstract

**Graphical Abstract:**
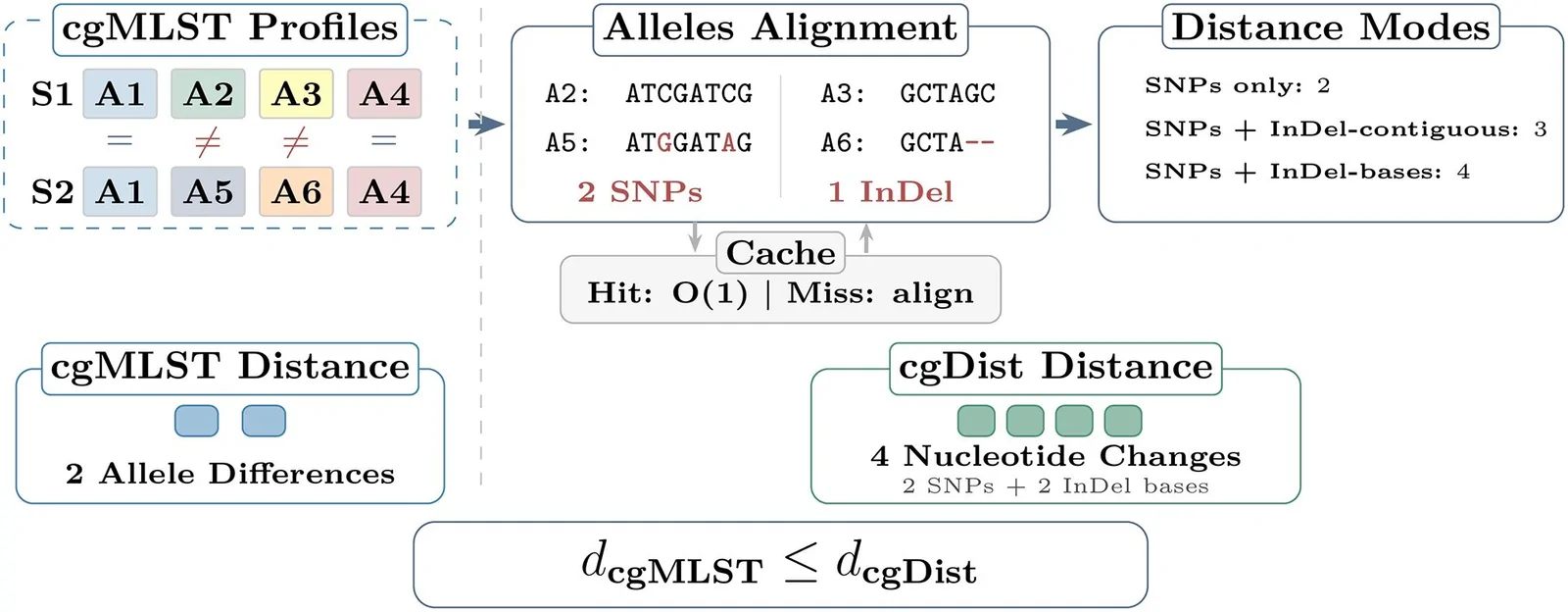

## Introduction

Bacterial genomic epidemiology has been transformed by the advent of next-generation sequencing technologies, enabling high-resolution analysis of pathogen transmission patterns and outbreak investigations [1, 2]. This revolution has been particularly impactful for foodborne pathogens such as *Listeria monocytogenes* and *Salmonella enterica*, where accurate source attribution is important for public health protection and outbreak control [3, 4].

Core genome multilocus sequence typing (cgMLST) is a widely adopted reference method for bacterial strain typing in surveillance and outbreak investigation workflows [5, 6]. This approach characterizes bacterial isolates using allelic profiles derived from conserved core genome loci, providing a standardized framework that balances discriminatory power with computational tractability [7, 8]. The method’s success stems from its ability to generate comparable results across different laboratories while maintaining sufficient resolution for epidemiological applications [6, 8].

Traditional cgMLST analysis compares isolates using allelic profiles, with pairwise distances commonly obtained by counting loci with different allele calls, corresponding to a Hamming-type distance over typing profiles [9]. While computationally efficient and widely adopted, this approach treats all allelic differences as equivalent units, regardless of the underlying nucleotide-level variation, potentially limiting resolution compared with single nucleotide polymorphism (SNP)-based analyses [10].

SNP-based methods generally provide higher phylogenetic resolution by examining individual nucleotide differences within genomic regions shared across all isolates (i.e. the core genome) [10–12]. These approaches process raw sequencing data or assembled genomes to construct SNP matrices that accurately reflect evolutionary relationships and transmission patterns. However, such methods require computational resources and complex bioinformatics pipelines, limiting their applicability for routine surveillance where rapid turnaround times are required [10, 13].

The computational challenges facing genomic surveillance systems are further compounded by Amdahl’s law, which demonstrates that the achievable speedup of parallel computation is bounded by the nonparallelizable fraction of the workload [14]. This limitation is particularly relevant for real-time surveillance applications, where processing thousands of isolates with rapid turnaround requirements demands both algorithmic efficiency and scalable performance characteristics [13].

Current genomic surveillance infrastructure faces a gap between the computational efficiency required for routine monitoring and the resolution necessary for accurate epidemiological inference. A practical solution would leverage precomputed allelic profiles to minimize computational overhead while incorporating nucleotide-level information to enhance discriminatory power, remaining independent of the specific allele calling methodology to ensure compatibility across different surveillance platforms and laboratory workflows. Such an approach would enable rapid integration of new isolates without genome reprocessing, thereby supporting timely cluster investigation and downstream follow-up analyses.

An additional complexity in outbreak cluster delineation arises from horizontal gene transfer and genetic recombination events, which can inflate genetic distances between epidemiologically related isolates and disrupt cluster boundaries [15, 16]. These genetic exchanges can cause closely related isolates to appear more distant than expected and can complicate phylogenetic interpretation and outbreak-cluster delineation [16, 17].

Here, we present cgDist, an algorithm that exposes nucleotide-level variation hidden within cgMLST allelic profiles. cgDist is designed as a complementary refinement applied selectively to clusters identified through standard cgMLST screening, enabling nucleotide-level characterization within existing genomic surveillance workflows. In addition, the unified cache architecture also supports exploratory companion analyses, including a candidate recombination-region flagging approach.

## Methods

**Terminology clarification:** The term ‘distances’ is used in a computational context in this manuscript and refers to genomic differences between pairs of genomes, rather than phylogenetic distances that represent evolutionary time. Additionally, ‘cgMLST distance’ refers to the traditional allelic Hamming distance calculation used in cgMLST analysis, where each allelic difference contributes one unit regardless of the underlying sequence variation.

### Algorithm design and implementation

#### cgDist algorithm: Conceptual framework

The fundamental concept behind cgDist is straightforward: instead of treating all genetic differences as equal units, we examine the actual DNA sequences using cgMLST data to count the precise number of nucleotide differences between bacterial samples. Traditional cgMLST analysis assigns each gene variant a unique identifier (like a barcode) and calculates distances by simply counting how many identifiers differ between samples—this is called allelic Hamming distance, which we use throughout this manuscript to refer to traditional cgMLST distance calculations. While this approach provides standardized and reproducible results, it cannot distinguish between alleles with minimal sequence variation and those with substantial nucleotide differences.

**Figure 1.**
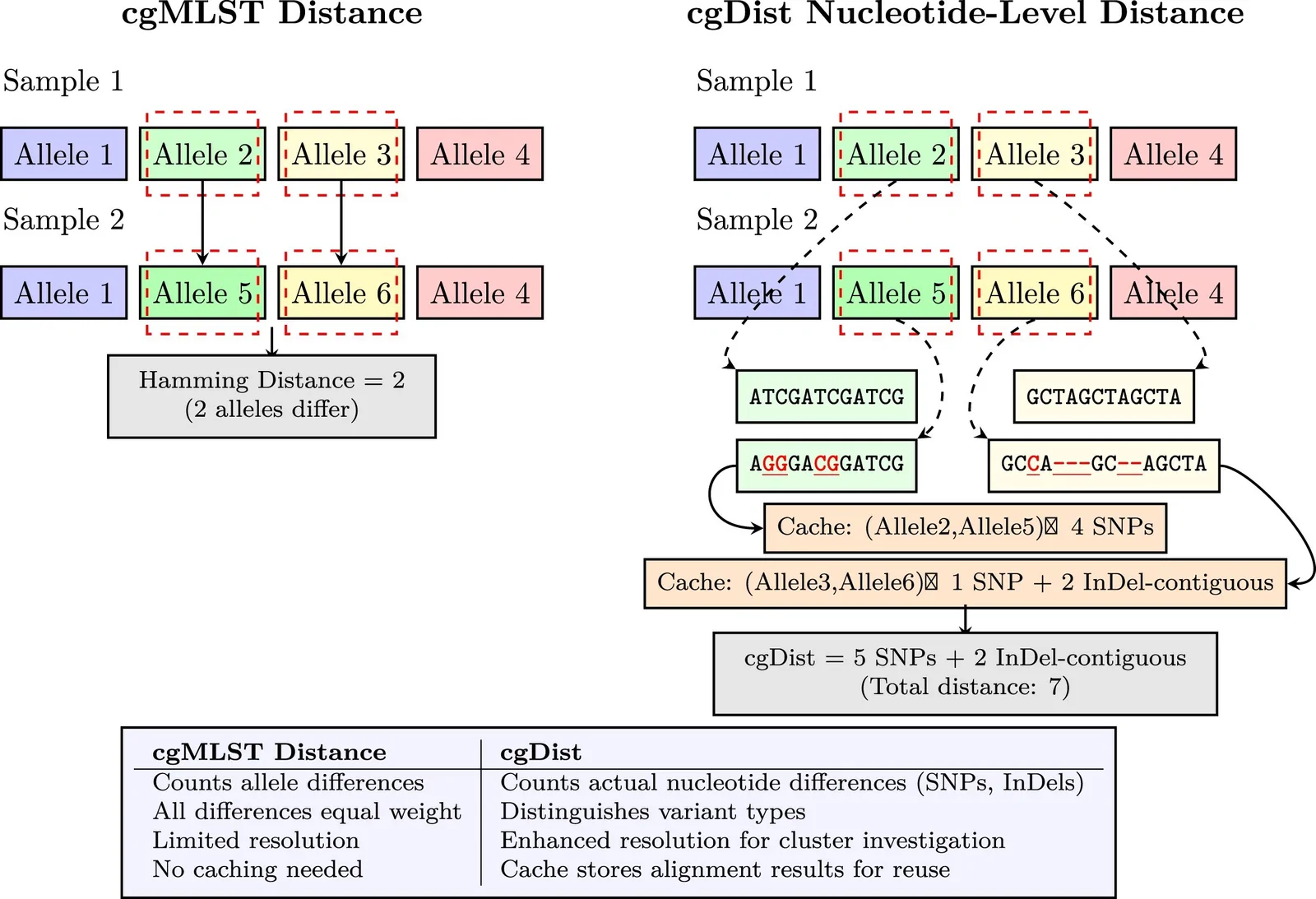
Comparison of distance calculation approaches in bacterial genomics. The figure shows two methods: cgMLST distance (left) counts genes with different allele identifiers, treating all differences equally (distance = 2 in this example). cgDist (right) examines the actual DNA sequences behind differing alleles to quantify both SNPs and contiguous indels, revealing that two allele differences actually represent 5 SNPs and 2 contiguous indels (total distance = 7). The cache system stores alignment results (orange boxes) for performance optimization, demonstrating how cgDist intuitively provides higher resolution than cgMLST distance (7 versus 2) for epidemiological investigations.

In contrast, when two samples have different barcodes for the same gene, cgDist retrieves the actual DNA sequences behind those barcodes and aligns them to count the exact number of SNPs and insertion–deletion events (InDels). For example, if traditional analysis shows ‘2 allele differences’ between two bacterial isolates, cgDist might reveal this actually represents ‘5 SNPs and 2 contiguous indels’—providing much finer resolution for distinguishing closely related outbreak strains from unrelated background isolates (Fig. 1). This enhanced precision is particularly relevant in food-safety investigations, where improved discrimination among closely related isolates can support source attribution analyses [3, 18], and in healthcare-associated investigations where additional genomic resolution may help distinguish potential transmission links from coincidental similarity [19].

#### Algorithm implementation

cgDist is implemented in Rust, leveraging the language’s guarantees of memory safety [20] and high-performance execution model, to compute nucleotide-level distances directly from cgMLST allelic profiles through sequence-level analysis. Unlike traditional gene-by-gene cgMLST comparisons [5, 6], which commonly count differing loci as equivalent units in Hamming-type profile distances [9], cgDist performs pairwise sequence alignment using the Needleman-Wunsch global alignment algorithm [21] via the Parasail library (parasail-rs v0.3.5 Rust bindings) [22] to quantify actual SNP and InDel differences between alleles. This approach provides enhanced genomic clustering resolution for surveillance applications while maintaining the computational advantages of working with cgMLST data, allowing incremental analysis without reprocessing entire datasets from raw sequencing data. Technical implementation details and debugging capabilities are described in Supplementary Methods.

##### Distance calculation modes

cgDist supports four complementary distance calculation modes, each designed for specific epidemiological applications. The **SNPs-only** mode counts SNPs exclusively, providing the finest resolution for highly similar isolates. The **SNPs + InDel-contiguous** mode combines SNPs with the number of maximal contiguous indel regions in the alignment, where each contiguous gap is counted once regardless of its length. The **SNPs + InDel bases** mode counts SNPs plus the total number of inserted or deleted nucleotides, providing comprehensive nucleotide-level divergence measurement. The **Hamming** mode computes traditional cgMLST allelic differences for baseline comparison and validation.

##### Unified distance cache architecture and memoization

cgDist implements a unified distance cache system that stores all alignment statistics (SNPs, InDel-contiguous, InDel bases) in a single cache entry, enabling alignment reuse across different analysis configurations for the same dataset. The system implements intelligent memoization by scanning allelic profiles to identify only unique allele pairs requiring alignment computation, eliminating redundant calculations through the symmetric property of pairwise distances. This design exploits repeated allele sharing in clonal or lineage-structured surveillance datasets [8], where many samples share identical alleles at multiple loci, to minimize computational redundancy.

This architecture provides performance benefits: (i) switching between distance calculation modes without recalculation, (ii) varying sample completeness thresholds ($\theta _s$) and loci completeness thresholds ($\theta _\ell$) requires only matrix filtering without recomputing alignments, (iii) incremental processing enables efficient integration of new isolates without reprocessing historical data, and (iv) cumulative cache benefits within species enable progressive performance gains as surveillance programs accumulate alignment data over time.

The cache employs a two-level architecture with hash-based keys for rapid lookup and compressed binary storage for efficient disk usage. Each cache entry maps a locus-allele pair tuple to comprehensive alignment statistics including SNP count, InDel-contiguous, InDel bases, and computation timestamp. Cache metadata tracks alignment parameters, hasher type, and compatibility information to prevent misuse across different configurations.

The cache architecture supports two operational modes: dataset-specific caching where only allele pairs present in the current analysis are computed and stored, and schema-complete caching where all possible allele pair combinations within a cgMLST schema are pre-computed independently of any specific dataset. While schema-complete mode would enable processing all allele sequences from schema FASTA files to generate exhaustive alignment statistics for all possible allele pairs, this comprehensive pre-computation approach requires substantial computational resources and storage capacity, and is superfluous for the objectives of the current study. Therefore, it is not explored here but reserved for future investigation.

##### Input data requirements and output formats

cgDist requires three primary inputs: (i) a cgMLST allelic profiles matrix representing the sample dataset, where rows correspond to genomic samples and columns to cgMLST loci, with each cell containing the hash identifier for the corresponding allele, (ii) a cgMLST schema consisting of FASTA files containing nucleotide sequences for all alleles, and (iii) the distance calculation mode specification. Allelic profile matrices can be generated with cgMLST allele-calling tools such as ChewBBACA [7], and the corresponding FASTA-based cgMLST schema may be created locally or obtained from public registries such as Chewie-NS [23] or EnteroBase [24]. cgDist requires only that the hash identifiers in the profile matrix correspond to the allele sequences in the schema, and is otherwise independent of the allele-calling tool and schema source. An optional cache file containing pre-computed alignment statistics can be provided to accelerate computation by reusing previously calculated distances.

The allelic profiles matrix serves as the computational roadmap and defines the sample dataset for analysis, with hash identifiers acting as keys to retrieve sequences from the schema. cgDist supports multiple hashing algorithms with a flexible architecture for custom hashers, requiring only that hash values establish bijective mapping between matrix entries and schema sequences.

cgDist generates multiple output formats: (i) symmetric distance matrices in standard formats (TSV, CSV, PHYLIP, NEXUS), (ii) updated cache files containing newly computed alignment statistics for future reuse, (iii) detailed computation logs with performance metrics and cache utilization statistics, (iv) cache files with embedded metadata documenting analysis parameters and data provenance, and (v) optional human-readable alignment details enabling researchers to manually inspect specific nucleotide differences contributing to distance calculations, validate alignment quality, and identify potential sequencing or assembly artifacts. All outputs maintain sample identifier consistency with the input matrix and support integration into downstream phylogenetic analysis workflows.

##### Core algorithm and execution flow

**Figure 2.**
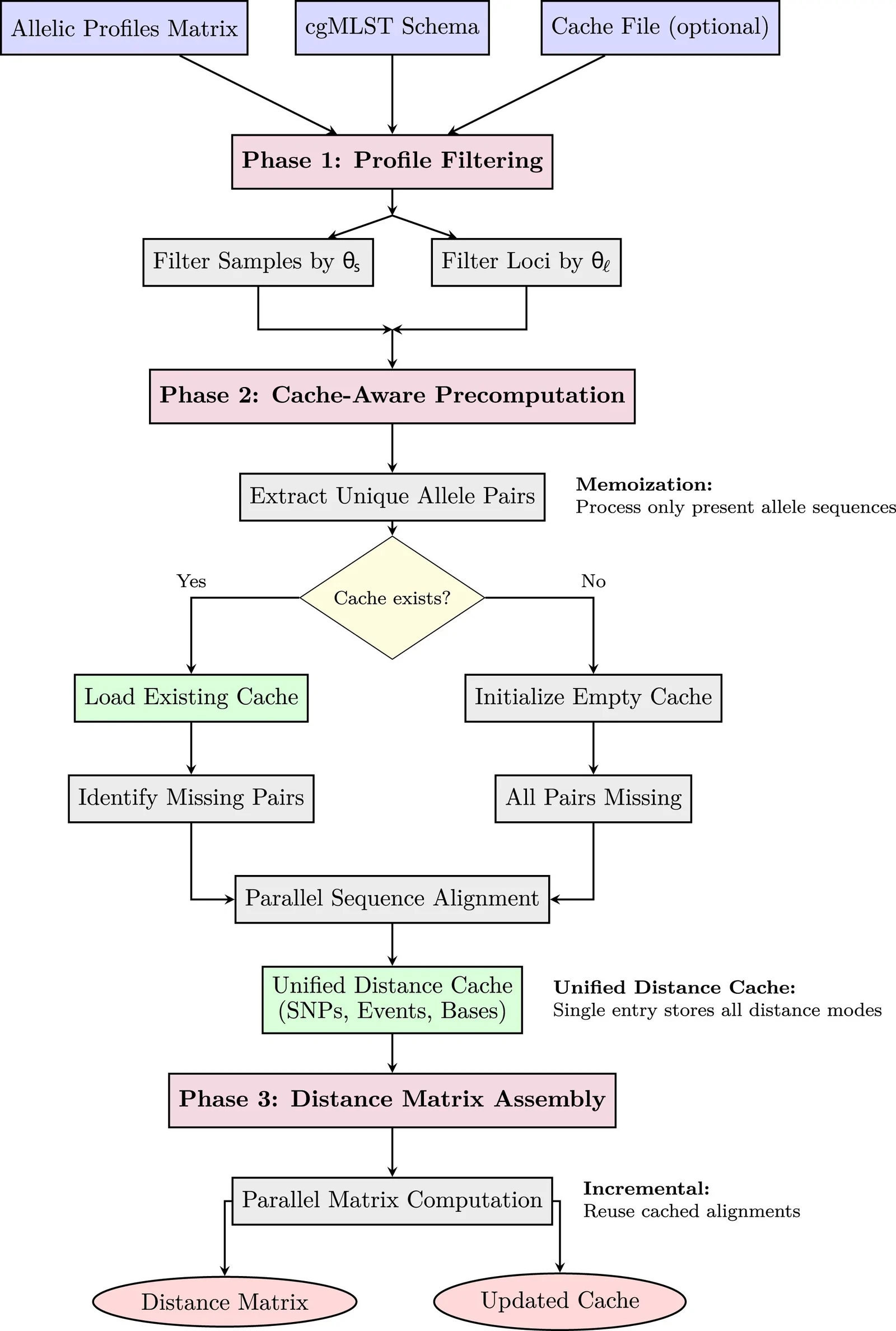
cgDist algorithm workflow showing the three-phase approach with cache-aware optimization. Phase 1 filters input profiles based on quality thresholds ($\theta _s$ for sample completeness, $\theta _\ell$ for locus completeness). Phase 2 implements memoization by extracting only unique allele pairs present in the dataset and leverages existing cache when available. Phase 3 assembles the distance matrix using parallel computation with cached alignment statistics. The unified distance cache architecture stores all distance calculation modes (SNPs, InDel-contiguous, InDel bases) in single entries, enabling rapid mode switching and incremental processing across surveillance runs.

The cgDist computation follows a three-phase approach designed for optimal performance and scalability (Algorithm 1, Fig. 2). Phase 1 performs profile filtering based on user-defined completeness thresholds for samples ($\theta _s$: minimum fraction of loci required per sample) and loci ($\theta _\ell$: minimum fraction of samples required per locus), enabling rapid exploration of different analytical stringency levels without recomputing alignments. To ensure epidemiologically meaningful results, the algorithm requires that sample pairs share a minimum number of loci (min_loci) before distances are computed, preventing unreliable estimates when too few loci are in common. Phase 2 implements cache-aware precomputation, extracting unique allele pairs and performing batch alignment only for missing cache entries. Phase 3 assembles the distance matrix using cached alignment statistics with parallel execution across sample pairs.

A design property of cgDist is that, in modes that include InDel contributions (SNPs+InDel-contiguous, SNPs+InDel-bases, or Hamming), the distance satisfies $d_{\mathrm{cgMLST}} \le d_{\mathrm{cgDist}}$ by construction, since differing alleles necessarily contribute either a SNP or a positive InDel count. In SNPs-only mode, however, this ordering may be violated for allele pairs whose differences are restricted to insertions/deletions (zero SNPs but different hashes), because such pairs contribute 0 to the cgDist distance while contributing 1 to the cgMLST allele-count distance. To preserve the ordering invariant in SNPs-only mode, cgDist provides an optional *Hamming fallback* option (–hamming-fallback). When enabled, allele pairs with zero SNPs but different hashes contribute *+1* to the cgDist distance. This *+1* contribution is a design-preserving placeholder rather than an inferred SNP count; in this context, a value of 1 does not represent a true nucleotide-level SNP difference. When the Hamming fallback is active, cgDist emits a warning to standard error indicating that these contributions should not be interpreted as true SNP differences. The fallback is disabled by default and must be explicitly enabled by users requiring the ordering invariant in SNPs-only mode.


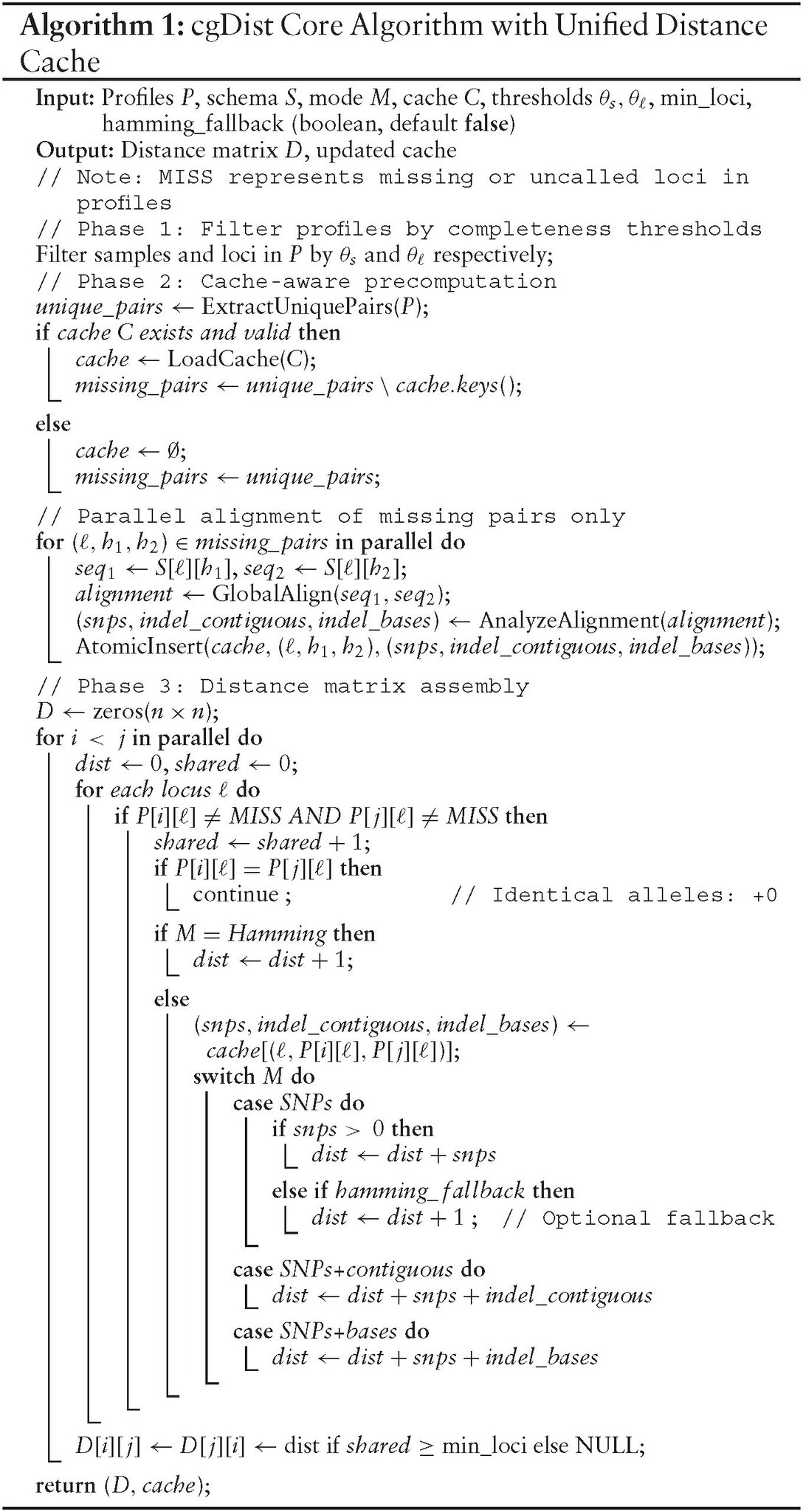


##### Distance calculation algorithm

The core distance calculation proceeds through sequence alignment analysis to extract nucleotide-level differences between allele pairs. For each unique allele pair identified in the profiles matrix, cgDist retrieves the corresponding nucleotide sequences from the schema and performs global sequence alignment using the Parasail library. The alignment results are then analyzed character-by-character to identify and classify genetic variants into three categories: SNPs, InDels (discrete gap regions), and InDel bases (total nucleotides involved in gaps).

The algorithm maintains running counters for each variant type while processing the alignment, ensuring consistent classification of detected variants. Gap-tracking logic identifies maximal InDel-contiguous regions in the alignment, such that each contiguous gap region is counted once regardless of its length. Different distance calculation modes selectively combine these counters to generate the final distance metrics according to the specified analysis requirements.

The detailed algorithm for analyzing pairwise sequence alignments and computing different distance metrics is provided in Supplementary Methods.

##### Alignment parameters and mode selection

For this study, we employed cgDist’s DNA-strict alignment mode to ensure conservative, high-confidence distance measurements. This approach builds upon established global alignment principles [21] with parameters optimized for bacterial genomic sequences (detailed parameters in Supplementary Methods).

#### Mathematical foundation: cgMLST-cgDist ordering

A foundational property of cgDist is that it is a conservative refinement of cgMLST allele-count distance:

Theorem 1 (cgMLST-cgDist Ordering). For any two genomic samples $S_i$ and $S_j$ analyzed using a mode that includes InDel contributions (SNPs+InDel-contiguous, SNPs+InDel-bases, or Hamming), or using SNPs-only mode with the optional Hamming fallback enabled:

\begin{eqnarray*}
d_{\mathrm{cgMLST}}(S_i, S_j) \le d_{\mathrm{cgDist}}(S_i, S_j),
\end{eqnarray*}

with equality, in SNPs-only mode, when each differing allele pair either contains exactly one SNP or triggers the Hamming fallback.

*Remark*. In SNPs-only mode without the Hamming fallback, the ordering invariant may be violated for allele pairs whose differences are confined to insertions/deletions, since such pairs contribute 0 to the cgDist distance while contributing 1 to the cgMLST allele-count distance. Users selecting this configuration therefore accept this trade-off in exchange for avoiding artificial inflation of SNP counts at indel-only loci.

The supporting lemma on per-locus contributions, the formal proofs, and the corollary describing the equality condition are provided in Supplementary Methods.

##### Computational complexity and performance

cgDist exhibits time complexity $O(n^2 \times s^2 \times l)$ for computing pairwise distances between *n* samples across *l* loci with maximum sequence length *s*. Cache optimization reduces this to $O(n^2 \times l)$ when alignment results are reused. Space complexity is $O(n^2 + l \times u^2)$ where *u* represents average unique alleles per locus, with the dominant term depending on dataset characteristics. Detailed complexity analysis including bounds, optimizations, and practical performance considerations is provided in Supplementary Methods.

##### Deterministic execution and reproducibility

cgDist guarantees deterministic results through hash-based allele identification, canonical cache keys using ordered hash pairs, deterministic sequence alignment, and parallel execution safety via read-only cache operations during matrix computation. Given identical inputs, cgDist produces identical distance matrices regardless of execution environment, thread count, or processing order. This deterministic behavior supports reproducible genomic surveillance workflows.

##### Extensibility and schema flexibility

cgDist implements a flexible architecture that enables adaptation to diverse cgMLST schemas and laboratory workflows. The system supports multiple hashing algorithms with a plugin system for implementing custom hashers to accommodate specialized applications or legacy compatibility requirements.

The flexible architecture enables laboratories to implement domain-specific hashing strategies, maintain compatibility with existing tools, or meet specific regulatory requirements while leveraging cgDist’s performance optimizations. Custom hashers can be designed for specialized organisms, experimental research applications, or integration with institutional bioinformatics pipelines.

Additionally, cgDist provides both command-line interfaces and programmatic APIs for integration into automated workflows, supporting multiple input/output formats and configuration management. This flexibility ensures compatibility with existing laboratory information management systems and enables seamless integration into diverse surveillance infrastructure without disrupting established analytical workflows.

### Experimental design and validation

#### Datasets and experimental setup

##### Validation datasets

We validated cgDist using four comprehensive datasets from the OHEJP BeONE project [25]: 1426 and 1874 *L. monocytogenes* isolates (referred to as Lm-1426 and Lm-1874 datasets, respectively) and 1434 and 1540 *S. enterica* isolates (referred to as Se-1434 and Se-1540 datasets, respectively) from European surveillance networks spanning multiple years and geographic regions. We used *L. monocytogenes* and *S. enterica* as representative models of contrasting population structures. *Listeria monocytogenes* provides a lineage-structured validation setting [8], whereas *S. enterica* provides a contrasting setting with broader genomic diversity across surveillance collections [24]. All four datasets were used for comprehensive validation, mathematical constraint verification, and performance benchmarking.

The selection of these bacterial species and dataset pairs provides complementary validation perspectives across different population genetic structures and sampling strategies. Together, these species provide contrasting diversity profiles in surveillance datasets, enabling assessment of cgDist performance across both relatively clonal collections and more genetically diverse sampling contexts. For each species, two datasets with distinct sampling designs were used: (i) datasets with comprehensive source attribution metadata (Lm-1426, Se-1540) representing sampling diversity across BeONE consortium partners, where isolate selection was determined by partner contributions and outbreak investigation priorities; and (ii) datasets without detailed source metadata (Lm-1874, Se-1434) representing species diversity from international public archives (ENA/SRA), providing broader coverage of genetic variation present in surveillance databases. This dual-dataset approach per species enables validation across both targeted surveillance collections and broader genomic diversity landscapes.

For epidemiological source prioritization analysis, we utilized the two datasets with comprehensive source attribution metadata: the 1426 *L. monocytogenes* dataset comprising isolates from clinical surveillance (76.4%), processing plant monitoring (14.1%), food samples (6.9%), and other sources, spanning 28 distinct sequence types with ST5 and ST6 predominant; and the 1540 *S. enterica* dataset including isolates from clinical (59.0%), environmental (3.1%), animal (5.8%), and other sources (32.1%), representing more than 75 sequence types with ST11, ST34, and ST19 most prevalent. The remaining two datasets (1874 *Listeria* and 1434 *Salmonella*) lack detailed source classification metadata and were therefore used exclusively for validation and performance testing.

Allelic profiles were generated using chewBBACA v3.3.10 [7] with the –hash-profiles crc32 parameter. For *L. monocytogenes*, we used the Institut Pasteur cgMLST schema (1748 loci) [8], and for *S. enterica*, we used the INNUENDO cgMLST schema [26] derived from the EnteroBase wgMLST schema [24], both available through ChewieNS [23] (accessed March 2024). For *S. enterica* analysis, we used the 3255-locus cgMLST subset of the INNUENDO wgMLST schema [26], to support compatibility with European food-safety WGS surveillance and reporting frameworks [27]. All assemblies met quality thresholds for reliable cgMLST analysis, and distance calculations satisfied the fundamental constraint $d_{\mathrm{cgMLST}} \le d_{\mathrm{cgDist}}$ across all datasets.

Mathematical validation, correlation analysis, and performance benchmarking were conducted using all available datasets. For epidemiological analyses including source prioritization and clustering resolution assessment, we applied a 98% completeness threshold ($\theta _s = 0.98$) requiring samples to have allele calls for at least 98% of the cgMLST loci. This threshold is consistent with established cgMLST quality control practices for *L. monocytogenes*, where high-quality profiles are expected to achieve very high cgMLST completeness [28]. For *S. enterica*, we applied the same stringent threshold, though this represents a more conservative filtering approach than typically required for *Salmonella* surveillance [29, 30]. This quality filtering is required for epidemiological analysis where incomplete allelic profiles could introduce noise in cluster investigation decisions.

##### Comparative analysis framework

For both species, we performed parallel distance calculations using cgmlst-dists for cgMLST distances [31] and cgDist in both SNPs-only and SNPs+InDel-contiguous modes. Both cgDist modes were selected to provide complementary perspectives on epidemiological source prioritization analysis: SNPs-only mode provides a focused analysis of nucleotide substitutions, while SNPs+InDel-contiguous mode provides a practical balance between resolution enhancement and epidemiological interpretability by capturing discrete contiguous indel regions without being influenced by the length heterogeneity that characterizes SNPs+InDel-bases mode. This framework enables direct assessment of resolution improvement and epidemiological utility across identical sample sets, with particular focus on epidemiologically relevant distance ranges (*≤*7 alleles for *L. monocytogenes* [8], *≤*10 alleles for *S. enterica* [29, 30]).

#### Validation and analysis methods

##### Mathematical validation and statistical analysis

We implemented systematic, schema-independent validation of the core design constraint $d_{\mathrm{cgMLST}} \le d_{\mathrm{cgDist}}$ by computing both distance types for all pairwise comparisons across diverse cgMLST schemas and bacterial species. This validation framework is inherently schema-independent, requiring no prior knowledge of locus selection, allelic diversity, or species-specific genetic architecture. Any constraint violations are flagged for detailed investigation, including examination of underlying allelic profiles and sequence alignments. The validation framework also confirms the correct implementation of the optional Hamming fallback mechanism (when enabled) for allele pairs with different hash identifiers but zero detected SNPs after sequence alignment.

The schema-independent nature of our validation approach ensures that the mathematical guarantees established for cgDist apply universally across any cgMLST scheme, regardless of the specific loci included, target organism, or laboratory implementation. This universality is achieved through the fundamental property that the ordering relationship is preserved at the sequence level for any pair of alleles, independent of their biological context or schema design principles.

##### Statistical correlation analysis

To assess whether cgDist maintains consistent relationships with cgMLST distances while providing enhanced resolution, we computed both Pearson correlation coefficients [32] and Spearman rank correlation [33] between distance methods. Pearson correlation measures linear relationships between the actual distance values, evaluating whether cgDist distances scale proportionally with cgMLST distances. Spearman rank correlation assesses the preservation of relative ordering between isolate pairs, which is critical for epidemiological clustering where rank-based relationships determine outbreak linkage decisions.

##### Multiscale clustering analysis framework

To evaluate the utility of cgDist across different epidemiological scales—from global population-level analysis to detailed cluster investigation—we propose a two-tier exploratory workflow intended to support future systematic comparison with established single-method surveillance practices. The two tiers are: (i) **Population screening**—initial cgMLST analysis to identify epidemiologically relevant clusters and potential outbreak groups; and (ii) **Cluster zoom analysis**—cgDist nucleotide-level distance calculation on selected clusters to enhance resolution and improve source attribution accuracy. An exploratory companion analysis for candidate recombination-region flagging enabled by the same cache architecture is described in Supplementary Methods.

**Tier 1: Global resolution assessment via single linkage clustering**

We first assessed the impact of cgDist on global clustering patterns using single linkage hierarchical clustering as a standardized analytical framework. This choice is consistent with distance-based approaches evaluated in recent interpipeline assessments of foodborne pathogen genomic surveillance, where single-linkage hierarchical clustering was among the clustering strategies assessed [25]. Single linkage clustering was performed using Python 3.11 with SciPy v1.11.4 [34] on all four datasets (Lm-1426, Lm-1874, Se-1540, Se-1434) comparing cgMLST distance against both cgDist modes (SNPs-only and SNPs+InDel-contiguous). For clustering congruence analysis using the adjusted rand index (ARI) [35], we evaluated a range of thresholds (1–15 alleles) to assess method agreement across different stringency levels. This method provides a systematic framework to evaluate how enhanced genomic resolution affects cluster detection at the population level.

The global analysis examines three critical aspects: (i) **Cluster resolution enhancement**—measuring how cgDist increases the number of detected clusters and changes cluster size distributions compared to cgMLST distance; (ii) **Cluster composition changes**—quantifying which isolates change cluster assignments and the epidemiological impact of reassignments; and (iii) **Clustering congruence**—evaluating consistency between methods using the ARI to quantify the proportion of samples experiencing cluster assignment changes.

Hierarchical clustering using the single linkage method was performed on cgMLST distance and cgDist matrices (both SNPs-only and SNPs+InDel-contiguous modes) at epidemiologically relevant operational thresholds (*≤*7 alleles for *L. monocytogenes* [8], *≤*10 alleles for *S. enterica* [29, 30]). All four datasets were filtered to 98% sample completeness threshold ($\theta _s = 0.98$).

The analysis quantifies the number of isolate pairs clustered together at a given threshold using cgMLST distance but separated by cgDist nucleotide-level analysis [36]. Whether such reassignments correspond to epidemiologically distinct events or simply reflect within-noise variation would require evaluation against ground-truth investigation records, which is outside the scope of the present study. Additionally, we analyze cluster-splitting patterns to identify large cgMLST-defined clusters that are subdivided into smaller subclusters by cgDist, thereby providing finer candidate–source ranking within clusters [18, 37].

**Tier 2: Food safety source prioritization via star clustering**

Following the global assessment, we implemented patient-centered source prioritization using a star-clustering topology as an analytical design choice. For each clinical isolate, we constructed a patient-centered star cluster using Python 3.11 and NetworkX v3.2 [38], where the patient serves as the central node and all nonpatient isolates within established epidemiological thresholds serve as directly connected candidate–source nodes. This design reflects the source-attribution logic of food-safety investigations, where clinical isolates are compared with food, environmental, or processing-plant isolates to prioritize plausible sources [3, 18].

In each star cluster, all candidate–source isolates lie within the specified threshold distance of the clinical isolate, creating well-defined sets of closely related isolates around each patient case and enabling source prioritization based solely on patient–source genetic distances.

Only patients with at least one candidate source within the epidemiological threshold can form a star cluster. Within each star cluster, potential sources are ranked by their genomic distance to the patient isolate using hierarchical tiebreaking: primary sorting by cgDist distance, secondary sorting by cgMLST distance for tied cgDist values, and tertiary lexicographic sorting by sample identifier for remaining ties. This hierarchical approach ensures that cgDist rankings preserve the fundamental epidemiological relationships established by cgMLST while adding nucleotide-level granularity, enabling prioritization of investigation efforts with lower-ranked sources receiving higher investigative priority.

The analysis utilized the 1426 *L. monocytogenes* and 1540 *S. enterica* datasets with comprehensive source classification metadata essential for distinguishing patients from potential food sources. Critical rank shifts are defined as patient–source relationships where sources ranked within the top three positions by cgMLST distance are displaced outside the top three positions by cgDist analysis. These shifts represent cases where nucleotide-level reordering could alter candidate–source prioritization in food-safety investigations [3].

##### Note on exploratory recombination-candidate flagging analysis

Beyond the four distance calculation modes, the cgDist cache architecture supports an additional exploratory companion analysis. When caches are enriched with allele sequence-length information (optional setting), the system can compute per-locus nucleotide-difference densities to flag candidate regions with unusually high within-allele divergence. We emphasize that this approach is a heuristic candidate-flagging procedure rather than a validated method for detecting recombination or horizontal gene transfer. Confirmation of flagged regions would require dedicated tools such as Gubbins [17], ClonalFrameML [39], or fastGEAR [40], and lies beyond the scope of the present analysis. Full algorithmic details, threshold-selection procedures, and analysis outputs for the four datasets are provided in Supplementary Methods.

##### Performance analysis and incremental processing

We conducted comprehensive scalability analysis using high-performance computing infrastructure and developed a Nextflow pipeline [41] for incremental processing validation. The pipeline simulates temporal arrival of isolates in surveillance scenarios, measuring computational performance, cache efficiency, and alignment reuse benefits as surveillance programs continuously integrate new genomic data. Detailed methodology for performance benchmarking, threading analysis, and the complete Nextflow pipeline architecture are described in Supplementary Results.

## Results

### Mathematical validity and consistency

#### Mathematical constraint validation

Validation confirmed that all distance calculations satisfied the fundamental design constraint established by the cgMLST-cgDist Ordering Theorem that cgDist values are greater than or equal to corresponding cgMLST distances: ($d_{\mathrm{cgMLST}} \le d_{\mathrm{cgDist}}$). Comprehensive testing across all four datasets totaling 4983 517 pairwise comparisons confirmed zero violations of the expected ordering relationships: $d_{\mathrm{cgMLST}} \le d_{\mathrm{cgDist}_{\mathrm{SNPs}}} \le d_{\mathrm{cgDist}_{\mathrm{SNPs+InDel-contiguous}}} \le d_{\mathrm{cgDist}_{\mathrm{SNPs+InDel-bases}}}$. This comprehensive testing validates both the theoretical algorithm design and practical implementation accuracy across diverse bacterial species, dataset sizes, and genetic diversity levels (Table 1).

**Table 1.** Correlation analysis between distance methods

Comparison	Lm-1426	Lm-1874	Se-1540	Se-1434
	(1016 025 pairs)	(1755 001 pairs)	(1185 030 pairs)	(1027 461 pairs)
	r/*ρ*	r/*ρ*	r/*ρ*	r/*ρ*
SNPs versus cgMLST	0.771/0.933	0.788/0.876	0.716/0.931	0.901/0.905
SNPs+InDel-contiguous versus cgMLST	0.771/0.933	0.789/0.880	0.716/0.931	0.901/0.905
SNPs+InDel-contiguous versus SNPs	1.000/1.000	1.000/1.000	1.000/1.000	1.000/1.000
SNPs+InDel-bases versus cgMLST	0.771/0.921	0.789/0.872	0.699/0.890	0.772/0.784
SNPs+InDel-bases versus SNPs	1.000/0.994	1.000/0.996	0.966/0.947	0.891/0.911
SNPs+InDel-bases versus SNPs+InDel-contiguous	1.000/0.994	1.000/0.996	0.966/0.949	0.893/0.914

All correlations are significant at *P* <.001. r = Pearson correlation coefficient, *ρ* = Spearman rank correlation coefficient.

**Note:** All datasets: 100% completeness threshold. Lm-1426, Lm-1874: *L. monocytogenes*. Se-1540, Se-1434: *S. enterica*.

Strong correlations between distance methods confirmed algorithmic consistency (Pearson 0.699–1.000, Spearman 0.784–1.000, all *P* <.001). Perfect correlations between SNPs+InDel-contiguous and SNPs modes reflect the additive nature of contiguous indels, while high correlations with cgMLST distances demonstrate that cgDist preserves fundamental epidemiological relationships while adding nucleotide-level granularity.

### Multiscale clustering analysis: from global screening to cluster expansion

#### Tier 1: Global resolution assessment via single linkage clustering

Single linkage clustering analysis on complete 98% filtered datasets reveals substantial variation in maximum cluster sizes and shows how cgDist systematically subdivides large clusters, thereby increasing discriminatory resolution within genetically related groups (Table 2). Maximum cluster sizes vary significantly across datasets: Lm-1426 exhibits the largest clusters (up to 201 samples with Hamming distance, 199 with cgDist methods), while Se-1434 shows more conservative clustering (maximum 31 samples with Hamming, 26–28 with cgDist). Lm-1874 and Se-1540 demonstrate intermediate patterns with maximum cluster sizes of 43 and 124–125 samples, respectively.

**Table 2.** Largest cluster subdivisions by cgDist methods across all datasets using single linkage clustering

Dataset	cgDist mode	cgMLST rank	cgMLST size	cgDist groups	cgDist subdivision
Lm-1426	SNPs-only	1	201	3	199+2*×*1
		2	169	3	167+2*×*1
		3	34	2	33+1*×*1
	SNPs+InDel-contiguous	1	201	3	199+2*×*1
		2	169	4	166+3*×*1
		3	44	2	43+1*×*1
		4	34	2	33+1*×*1
		5	17	3	15+2*×*1
Lm-1874	SNPs-only	1	39	2	38+1*×*1
	SNPs+InDel-contiguous	1	39	2	38+1*×*1
		2	11	2	[6,5](d*≥*8)
Se-1540	SNPs-only	1	125	2	124+1*×*1
		2	122	6	117+5*×*1
		3	75	6	70+5*×*1
		4	72	3	70+2*×*1
		5	44	3	42+2*×*1
	SNPs+InDel-contiguous	1	125	2	124+1*×*1
		2	122	7	116+6*×*1
		3	75	11	65+10*×*1
		4	72	6	67+5*×*1
		5	44	4	41+3*×*1
Se-1434	SNPs-only	1	31	4	28+3*×*1
		2	30	4	27+3*×*1
		3	18	2	17+1*×*1
		4	15	2	14+1*×*1
		5	12	6	[6,2](d*≥*11)+4*×*1
	SNPs+InDel-contiguous	1	31	7	25+6*×*1
		2	30	5	26+4*×*1
		3	18	2	17+1*×*1
		4	15	2	14+1*×*1
		5	12	6	[6,2](d*≥*11)+4*×*1

Single linkage clusters identified by cgMLST distance at epidemiological thresholds (≤7 for *Listeria*, ≤10 for *Salmonella*) are subdivided by cgDist into distinct genetic groups. Rankings show the five largest original cgMLST clusters (by size) that were subdivided by cgDist analysis for each dataset and cgDist mode. cgDist Subdivision uses bracket notation for multisample clusters with intercluster distances followed by singleton count (e.g. ”[5,2](d≥12)+4×1” indicates two clusters of 5 and 2 samples separated by ≥12 distance, plus 4 singletons).

**Note:** 98% completeness datasets. Lm-1426 (*n* = 1381), Lm-1874 (*n* = 1824): *L. monocytogenes*. Se-1540 (*n* = 1446), Se-1434 (*n* = 1192): *S. enterica*. Shows clusters *≥*10 samples subdividing into multiple groups.

**Species-specific subdivision patterns:** Analysis reveals distinct patterns between bacterial species. *Listeria monocytogenes* demonstrates conservative subdivision: the largest cluster in Lm-1426 (201 samples, all within *≤*7 alleles) subdivides into 199 closely related samples plus 2 genetic outliers exceeding 8 SNPs from the main group, potentially representing contamination, mislabeling, or convergent evolution. The 169-sample cluster similarly shows minimal fragmentation. Lm-1874 provides evidence of differential resolution between cgDist modes, with an 11-sample cluster remaining intact under SNPs-only analysis but subdividing into two distinct groups of 6 and 5 samples (separated by *≥*8 SNPs) under SNPs+InDel-contiguous analysis. *Salmonella enterica* exhibits more extensive fragmentation reflecting higher genetic diversity: a 75-sample cluster in Se-1540 subdivides into 11 distinct genetic groups (65 main + 10 singletons), consistent with genetically distinct subclusters that are not resolved by allelic distance alone. Se-1434 demonstrates true multicluster subdivision where clusters split into multiple subclusters (e.g. one 8-sample cluster splitting into subclusters of 6 and 2 samples separated by *≥*11 SNPs, plus 4 singleton outliers), indicating genetic subpopulations separated by epidemiologically significant distances.

**Figure 3.**
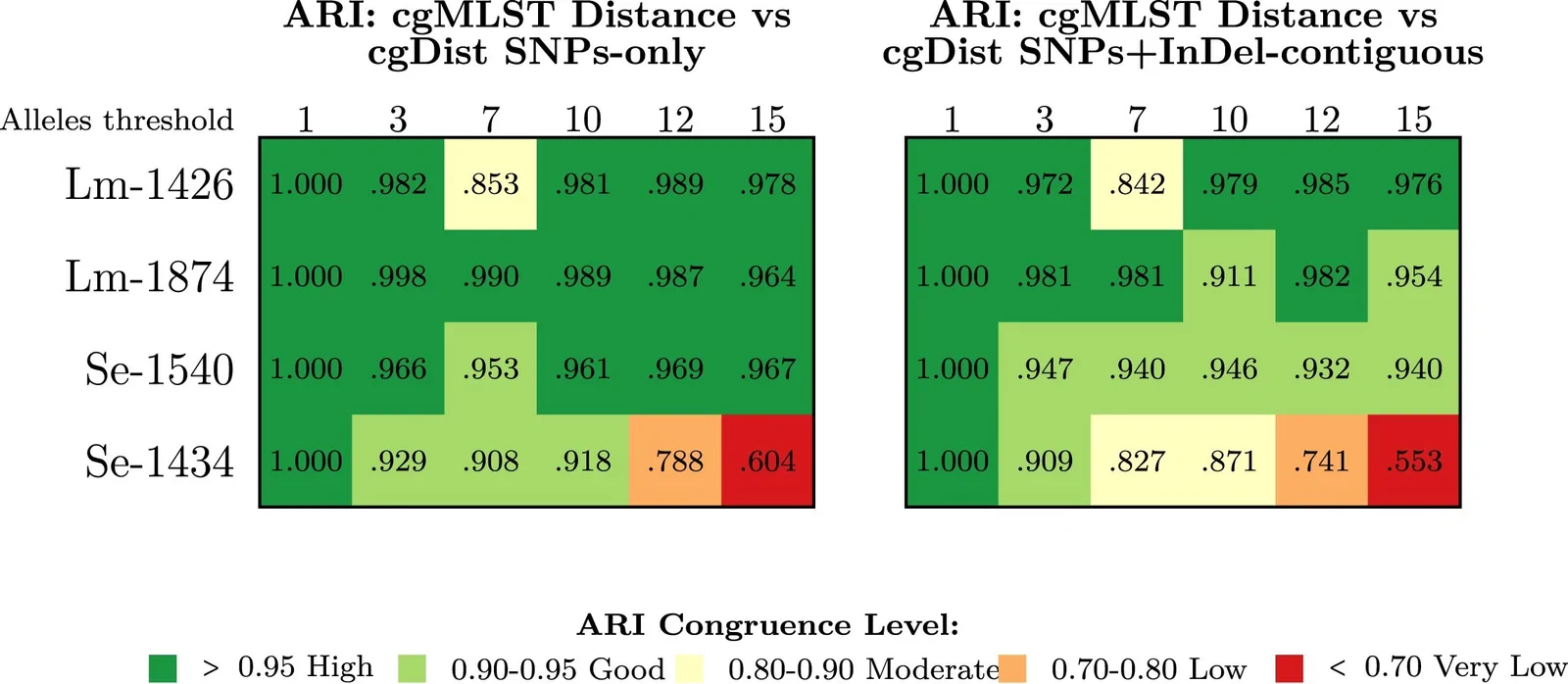
Threshold-dependent ARI congruence analysis between cgMLST distance and cgDist modes using single linkage clustering. Heatmaps display ARI values measuring congruence between single linkage clustering results at selected critical thresholds (1, 3, 7, 10, 12, 15 alleles). Each cell represents ARI between traditional cgMLST distance single linkage clustering and enhanced cgDist single linkage clustering at the specified threshold. Color intensity indicates congruence level: dark green (high congruence, ARI *> * 0.95), medium green (good congruence, 0.90–0.95), yellow (moderate changes, 0.80–0.90), orange (low congruence, 0.70–0.80), red (substantial restructuring, *< * 0.70). Left panel compares cgMLST distance versus SNPs-only mode; right panel compares cgMLST distance versus SNPs+InDel-contiguous mode. *Listeria monocytogenes* demonstrates consistently high congruence across thresholds, while *S. enterica* Se-1434 exhibits progressive degradation culminating in substantial restructuring at higher thresholds (ARI 0.553–0.604).

**Clustering congruence:** Despite resolution enhancement, clustering methods showed high congruence as measured by ARI values at epidemiological thresholds (Table 3 and Fig. 3). Lm-1426 and Lm-1874 datasets achieved ARI values of 0.842–1.000, indicating that cgDist enhances resolution while preserving most existing cluster relationships. Se-1540 and Se-1434 datasets showed lower but still substantial congruence (ARI 0.553–1.000), reflecting the greater restructuring potential in these more genetically diverse datasets. These ARI values demonstrate that cgDist provides enhanced discrimination while preserving most cgMLST-defined cluster relationships.

**Table 3.** Clustering composition changes: how cgDist affects cluster definition using single linkage clustering

	Baseline	SNPs-only mode			SNPs+InDel-contiguous Mode		
Dataset	cgMLST clusters	Clusters	Split	Changed	Clusters	Split	Changed
*Listeria monocytogenes* **(*≤*7 alleles threshold)**							
**Lm-1426**	505	513 (+8)	6	413 (29.9%)	520 (+15)	11	503 (36.4%)
**Lm-1874**	1132	1140 (+8)	8	72 (3.9%)	1148 (+16)	15	105 (5.8%)
*Salmonella enterica* **(*≤*10 alleles threshold)**							
**Se-1540**	563	607 (+44)	28	662 (45.8%)	628 (+65)	35	693 (47.9%)
**Se-1434**	808	845 (+37)	23	205 (17.2%)	860 (+52)	30	231 (19.4%)

Numbers in parentheses show additional clusters compared to cgMLST distance baseline. Split values indicate the number of cgMLST distance clusters identified by single linkage clustering that were subdivided by cgDist analysis. Changed values show the number and percentage of samples residing in clusters that were subdivided by cgDist relative to the cgMLST distance baseline.

**Note:** 98% completeness datasets. Lm-1426 (*n* = 1381), Lm-1874 (*n* = 1824): *L. monocytogenes*. Se-1540 (*n* = 1446), Se-1434 (*n* = 1192): *S. enterica*. Single linkage clustering at species-specific thresholds.

**Clustering composition changes:** Clustering composition analysis reveals the impact of enhanced genomic resolution on cluster definition across all validation datasets (Table 3). Relative to the cgMLST baseline, cgDist modifies cluster boundaries with dataset-specific patterns reflecting the genetic diversity present in each sample collection. For *L. monocytogenes*, cgDist provides enhancement that preserves most existing epidemiological associations while refining ambiguous cases. The Lm-1426 dataset shows substantial clustering changes (29.9% samples in SNPs-only, 36.4% in SNPs+InDel-contiguous mode), while Lm-1874 demonstrates more conservative changes (3.9% and 5.8%, respectively), indicating dataset-specific genetic diversity impacts. For *S. enterica*, cgDist reveals hidden diversity within cgMLST distance clusters, reflecting the species’ higher genetic diversity. The Se-1540 dataset demonstrates significant subdivision potential with 45.8% samples changing assignments in SNPs-only mode and 47.9% in SNPs+InDel-contiguous mode. The Se-1434 dataset shows more moderate changes (17.2% and 19.4%, respectively), demonstrating variable genetic diversity across datasets. The systematic increase in cluster numbers, combined with dataset-specific change patterns, indicates that cgDist primarily splits existing clusters rather than randomly reassigning isolates, targeting epidemiologically ambiguous relationships where nucleotide-level analysis provides clearer discrimination.

#### Tier 2: Food safety source prioritization via star clustering

Our multiscale analysis framework demonstrates cgDist’s application: initial cluster identification using single linkage clustering followed by targeted source prioritization using star clustering methodology. In this patient-centered setting, star clustering provides a practical and interpretable framework for evaluating the enhanced resolution provided by cgDist within epidemiologically relevant investigation scenarios.

**Star clustering implementation:** Initial screening using cgMLST distance on quality-filtered datasets (98% completeness threshold) identified 264 *Listeria* clusters from 1070 total clinical samples (Lm-1426 dataset) and 136 *Salmonella* clusters from 847 total clinical samples (Se-1540 dataset). These clusters represent the starting point for multiscale analysis—each cluster becomes a focused unit where cgDist expands internal resolution to reveal hidden genetic relationships (Table 4).

**Table 4.** Median cluster sizes across distance calculation methods using star clustering methodology on 98% completeness-filtered datasets

		Median cluster size (range)		
Dataset	Clinical samples	cgMLST distance	cgDist SNPs-only	cgDist SNPs+InDel-contiguous
Lm-1426	1070	2 (2–91)	2 (2–89)	2 (2–87)
Se-1540	847	34 (2–100)	54 (2–89)	68 (2–87)

Star clustering performed using patient-centered methodology with epidemiological thresholds of ≤7 alleles for Listeria and ≤10 alleles for Salmonella. Clusters are reconstructed independently under each metric at the same threshold, and patients retaining no source within the threshold are excluded; the number of clusters therefore differs between columns (Lm-1426: 264, 260, 260; Se-1540: 136, 124, 117) and medians are not directly comparable across columns.

**Note:** 98% completeness datasets. Star clustering analysis limited to datasets with comprehensive source attribution metadata (Lm-1426 and Se-1540). Other datasets (Lm-1874, Se-1434) lack detailed source classification required for patient-centered clustering methodology.

Within each identified cluster, cgDist’s enhanced resolution expands our understanding of source–patient relationships, revealing priority reorderings invisible at the allelic level (Table 5).

**Table 5.** Priority reordering impact across cgDist modes for datasets with source attribution metadata

		cgDist mode	
Species	Metric	SNPs-only	SNPs+InDels
*Listeria monocytogenes*	Patients with sources	264	264
	Clusters with reordering	68 (25.8%)	82 (31.1%)
	Rank 1 changes	0 (0.0%)	0 (0.0%)
	Top 3 changes	1 (0.4%)	2 (0.8%)
	Top 10 changes	17 (6.4%)	26 (9.8%)
	Average rank shift	3.39	3.60
	Maximum rank shift	74	75
	Most extreme shift	Lm_0114: 15*→*89	Lm_0114: 15*→*90
	Critical rank shifts	1	2
*Salmonella enterica*	Patients with sources	136	136
	Clusters with reordering	76 (55.9%)	82 (60.3%)
	Rank 1 changes	4 (2.9%)	4 (2.9%)
	Top 3 changes	16 (11.8%)	16 (11.8%)
	Top 10 changes	41 (30.1%)	43 (31.6%)
	Average rank shift	6.66	6.99
	Maximum rank shift	81	81
	Most extreme shift	Se_0439: 87*→*6	Se_0439: 87*→*6
	Critical rank shifts	16	16

Priority changes calculated between cgMLST distance and cgDist rankings using hierarchical tiebreaking (SNPs → cgMLST → Sample ID). Critical rank shifts: sources ranked 1–3 by cgMLST distance displaced to >3 by cgDist (loss of top priority). Top three changes: sources remaining in top three but changing position.

**Note:** 98% completeness datasets. Analysis limited to datasets with comprehensive source attribution metadata: Lm-1426 (*L. monocytogenes*) and Se-1540 (*S. enterica*). Thresholds: *≤*7 alleles (*Listeria*), *≤*10 (*Salmonella*).

*Listeria monocytogenes* clusters show moderate internal reorganization from SNPs-only (25.8% clusters affected) to SNPs+InDel-contiguous mode (31.1% clusters affected), demonstrating that even within small, tight clusters, cgDist expands resolution to differentiate closely related sources. Remarkably, despite this internal expansion, top source stability is maintained with no rank 1 changes, indicating that cgDist refines rather than disrupts primary epidemiological associations.

*Salmonella enterica* clusters exhibit more extensive internal restructuring from SNPs-only (55.9% clusters affected) to SNPs+InDel-contiguous mode (60.3% clusters affected). This greater sensitivity to cluster expansion reflects the species’ higher genetic diversity, where cgDist reveals substantial hidden variation within cgMLST-defined clusters. The consistent moderate top source changes (2.9% rank 1 changes in both modes) demonstrate that cluster expansion can alter cluster investigation priorities.

The magnitude of priority changes revealed consistent patterns across cgDist modes with species-specific amplification effects. *Listeria* demonstrated relatively stable rank shift magnitudes between SNPs-only (average 3.39 positions, maximum 74) and SNPs+InDel-contiguous modes (average 3.60 positions, maximum 75), indicating minimal but measurable additive impact from contiguous indels on source prioritization. *Salmonella* showed comparable stability between modes, with SNPs-only (average 6.66 positions, maximum 81) and SNPs+InDel-contiguous (average 6.99 positions, maximum 81) exhibiting similar reordering magnitudes, suggesting that species-specific genetic diversity patterns, rather than InDel inclusion, primarily drive prioritization differences. The most substantial reordering involved patient Se_0003 (Fig. 6), where 11 sources were completely tied at 1 allele difference by cgMLST. cgDist revealed that source Se_0695 had only 1 SNP while other tied sources had 2–7 SNPs, ranking it as the closest candidate under the nucleotide-level distance metric (rank 1). Simultaneously, sources initially ranked 1–2 by cgMLST were demoted to ranks 9–11, having 6-7 SNPs despite identical allelic distance, demonstrating how cgDist reveals nucleotide-level variation that can substantially modify source prioritization even among apparently equivalent candidates.

**Figure 4.**
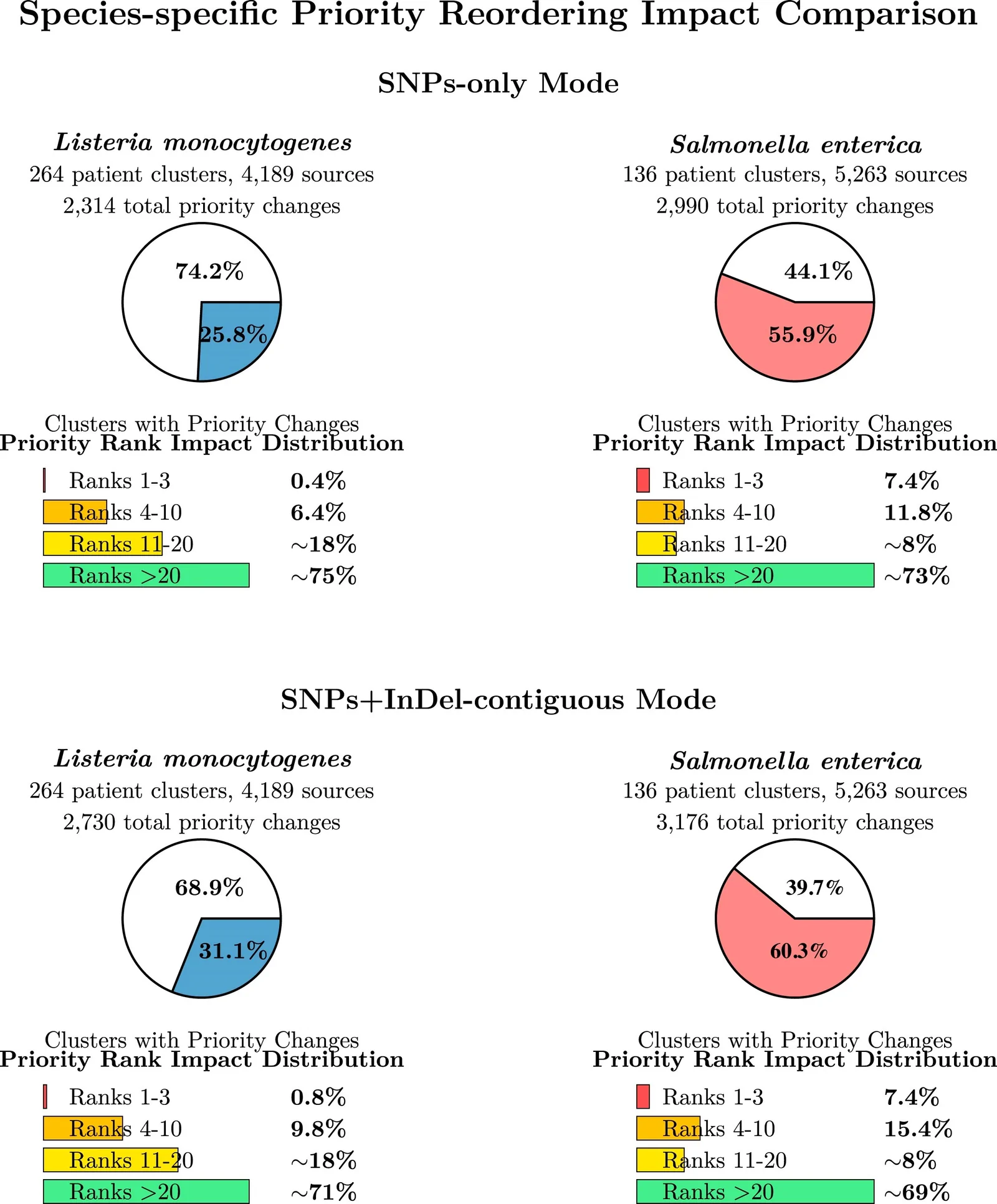
Direct comparison of species-specific source priority reordering impact between cgDist modes. Top panel shows SNPs-only mode analysis, bottom panel shows SNPs+InDel-contiguous mode analysis. **Pie charts**: in each panel, the coloured slice (blue for *L. monocytogenes*, pink for *S. enterica*) represents the proportion of patient clusters in which source-priority reordering occurred between cgDist and cgMLST distance methods; the white slice represents clusters with no reordering. *Listeria monocytogenes* shows moderate increases in reordering, ranging from 25.8% to 31.1% of affected clusters, with minimal impact on top-three source prioritization (0.4%–0.8%). *Salmonella enterica* exhibits substantial reordering in both modes (55.9%–60.3%) with consistent high top-three priority impact (7.4% both modes). Species-specific sensitivity patterns to InDel-contiguous inclusion are clearly visible through vertical comparison.

Critical rank shifts analysis revealed consistent patterns across cgDist modes, with species-specific thresholds for epidemiological significance (Fig. 4). *Listeria* showed minimal critical displacement increases from SNPs-only (one critical shift) to SNPs+InDel-contiguous mode (two critical shifts), maintaining relatively stable top-priority source identification. *Salmonella* demonstrated identical critical shift counts across both modes (16 critical shifts each), indicating that primary priority reordering occurs at the SNPs level rather than through contiguous indel inclusion. These findings suggest that while InDel-contiguous provide additional granularity for distance calculations, the fundamental epidemiological priorities for cluster investigations are established primarily through SNPs-level differences, with species-specific sensitivity patterns determining the magnitude of investigative impact.

**Figure 5.**
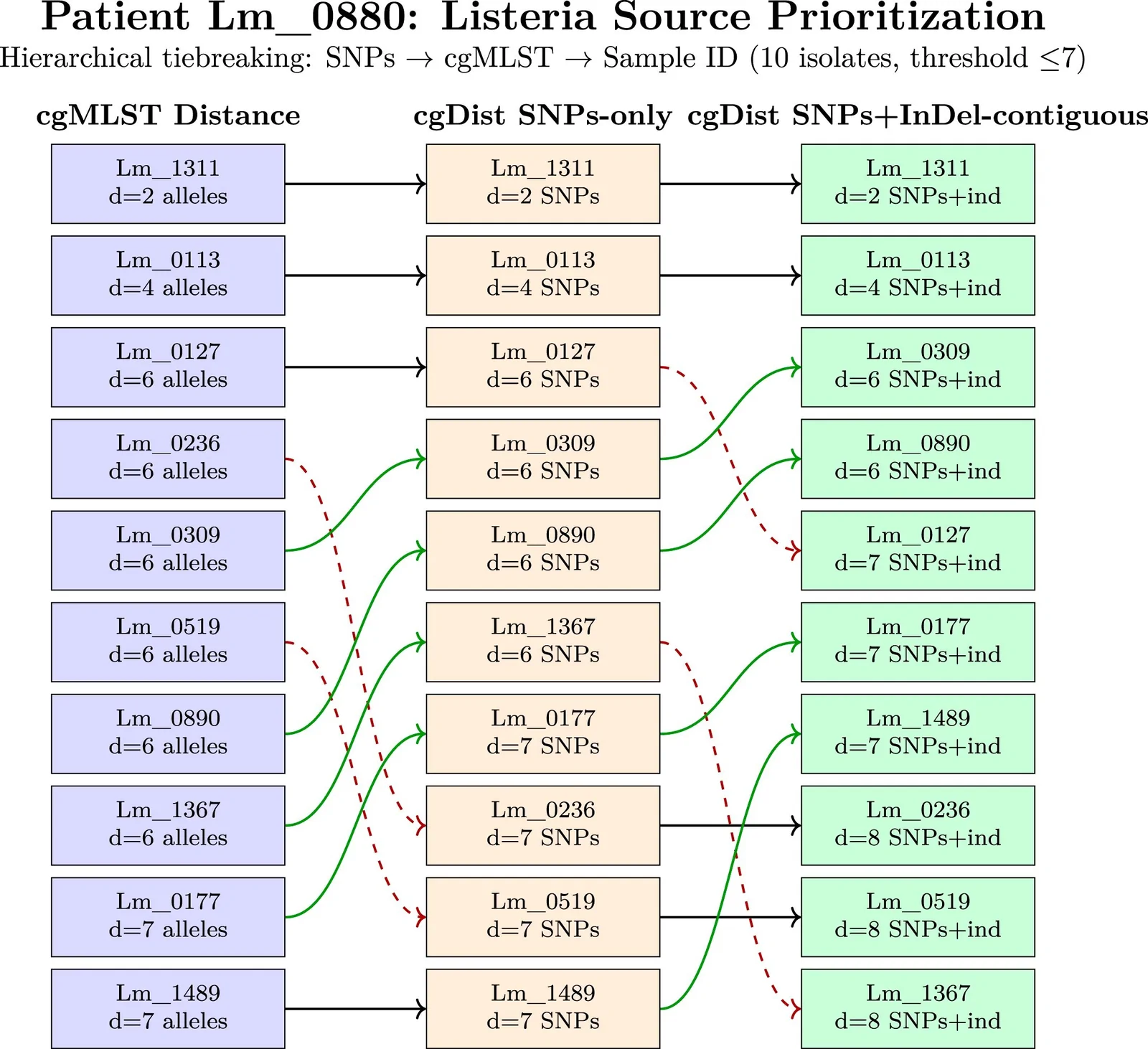
*Listeria monocytogenes* source prioritization comparing cgMLST distance versus both cgDist modes using hierarchical tiebreaking (SNPs *→* cgMLST *→* Sample ID). Patient Lm_0880 demonstrates substantial reordering between methods, with notable rank changes from cgMLST distance to SNPs-only and further movements to SNPs+InDel-contiguous, illustrating how different types of nucleotide-level variation contribute distinct information for *Listeria* source attribution.

**Figure 6.**
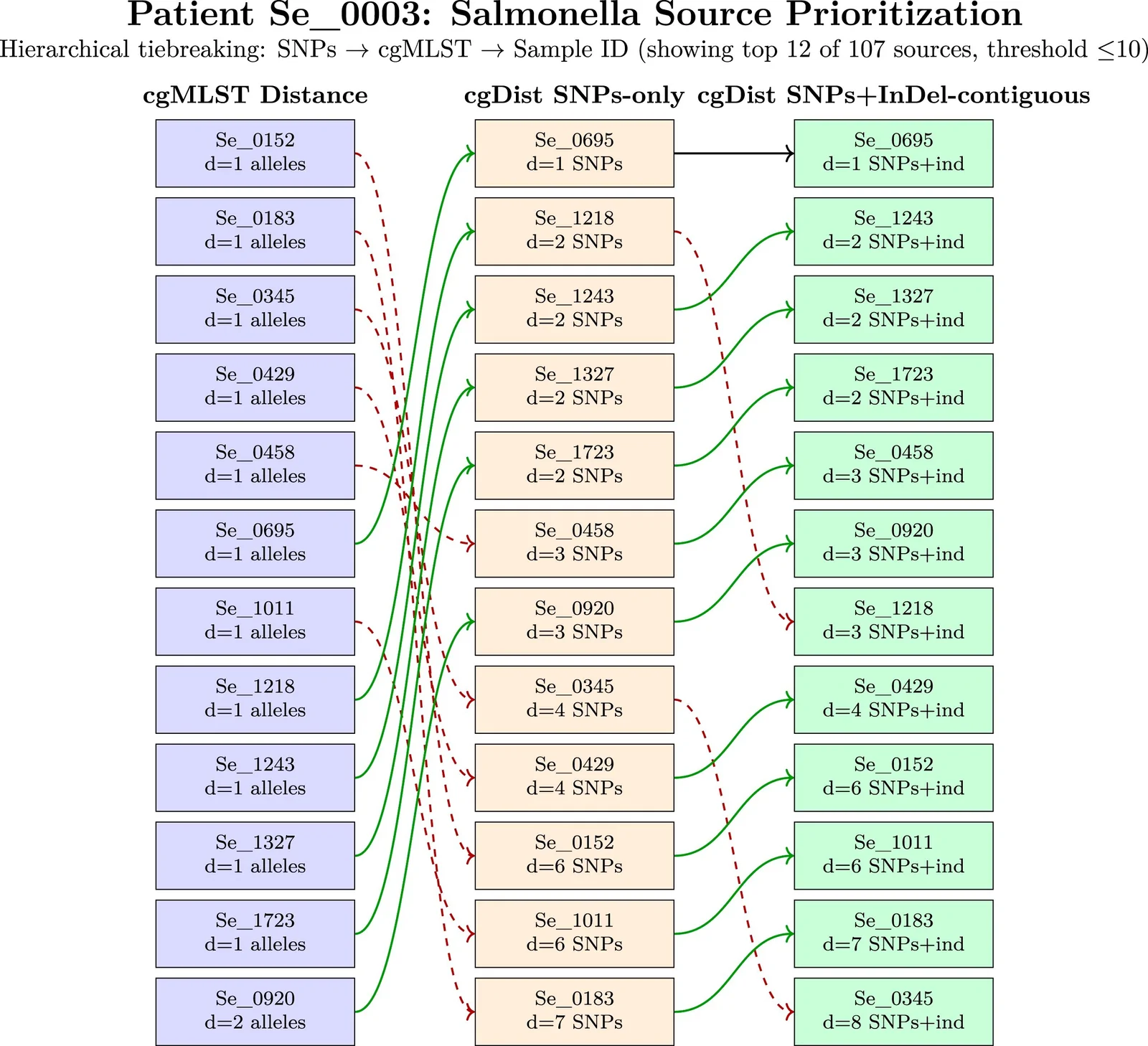
*Salmonella enterica* source prioritization comparing cgMLST distance versus both cgDist modes using hierarchical tiebreaking (SNPs *→* cgMLST *→* Sample ID). Patient Se_0003 (107 total sources) demonstrates extensive reordering between methods, with source Se_0695 advancing from rank 6 to rank 1 in both cgDist modes, while multiple highly ranked cgMLST distance sources are substantially demoted, illustrating how different types of nucleotide-level variation provide distinct discriminatory information for *Salmonella* source attribution.

**Source prioritization reordering:** Comparative analysis of cgDist modes revealed systematic patterns of candidate–source reordering across both SNPs-only and SNPs+InDel-contiguous calculation methods when compared to cgMLST distance methods (Figs 5 and 6).

Species-specific source-prioritization patterns emerged from the comparative analysis. *Listeria monocytogenes* demonstrated conservative but consistent reordering across both cgDist modes, with minimal critical rank shifts in SNPs-only mode (one patient–source relationship, 0.4% of clusters) increasing to modest levels in SNPs+InDel-contiguous mode (two patient–source relationships, 0.8% of clusters). The relatively homogeneous genetic structure of *Listeria* populations results in subtle but meaningful priority adjustments rather than extensive rank changes, indicating that both cgDist modes provide enhanced discrimination while maintaining epidemiological coherence. The incremental difference from SNPs-only to SNPs+InDel-contiguous mode suggests that contiguous indels contribute additional discriminatory power for source prioritization in *Listeria* investigations.

*Salmonella enterica* revealed more substantial source-prioritization reordering across both cgDist modes, reflecting the species’ higher genetic diversity. Both SNPs-only and SNPs+InDel-contiguous modes identified identical numbers of critical rank shifts (16 patient–source relationships each, 11.8% of clusters), indicating that SNPs-level analysis provides the primary discriminatory improvement for *Salmonella* source prioritization. The consistency across modes indicates that nucleotide-level analysis can substantially refine candidate–source ranking in genetically diverse bacterial populations, with SNPs contributing most of the discriminatory power and contiguous indels providing additional refinement.

Cross-mode analysis revealed that the impact of cgDist on source prioritization arises from systematic enhancement of genetic-resolution patterns rather than from isolated ranking corrections. Both cgDist modes consistently differentiate sources that appear similarly ranked under cgMLST distance but exhibit more distinct genetic relationships following nucleotide-level analysis. The most substantial reordering scenarios involve sources initially ranked within the top three positions by cgMLST distance but reassigned to lower-priority positions following cgDist analysis, thereby supporting more resolved candidate–source ranking during cluster investigations.

An exploratory companion analysis for recombination-candidate flagging applied to the same datasets, including its empirical *ρ* distribution and per-dataset output, is reported in Supplementary Methods (algorithm and threshold-selection procedure) and Supplementary Results (per-dataset flagging results).

### Performance characteristics and deployment validation

cgDist demonstrates dataset-dependent scalability patterns that correlate with genetic diversity rather than dataset size, achieving optimal parallel performance at 16–32 threads for high-diversity populations and near-linear scaling for low-diversity datasets. The unified cache architecture provides substantial performance improvements for multimode workflows, with cache reuse delivering up to 14*×* throughput improvements and 94% time reduction across analysis modes. Comprehensive performance analysis, scalability benchmarks, and memory efficiency evaluation are detailed in Supplementary Results.

### Surveillance pipeline applications

cgDist has been successfully integrated into the GENPAT bioinformatics platform alongside the SPREAD surveillance dashboard [42], contributing to real-time epidemiological analysis within an operational surveillance workflow. Incremental processing validation confirms that cgDist’s cache-aware design enables practical deployment of nucleotide-level genomic surveillance at the scale and speed required for modern public health practice. Detailed performance results from production surveillance integration and incremental processing validation are provided in Supplementary Results.

## Discussion

### Methodological innovation and validation

cgDist provides finer resolution than allele-count distance by leveraging within-allele nucleotide variation while reusing the standardized cgMLST inputs already generated in surveillance laboratories. Validation across all four datasets (Table 1) confirmed perfect preservation of the ordering constraint $d_{\mathrm{cgMLST}} \le d_{\mathrm{cgDist}}$ and strong correlations between distance methods, indicating that nucleotide-level refinement remains consistent with cgMLST-derived genetic relationship structure while providing additional discriminatory resolution.

The unified cache architecture represents a significant departure from traditional approaches that precompute entire schema combinations. By processing only allele pairs present in the current dataset and storing comprehensive alignment statistics in reusable cache entries, cgDist achieves computational efficiency comparable to cgMLST while providing nucleotide-level granularity.

### Tier 1–2 validation: The cluster zoom paradigm

Our multitier validation framework demonstrates the behaviour of cgDist across different surveillance contexts, with the most significant practical contribution emerging from its ability to ‘zoom into’ specific clusters for detailed investigation. The Tier 1–2 analysis intentionally focuses on distance-based clustering performance within cluster-investigation workflows, where enhanced SNP-level resolution can provide additional epidemiological discrimination without requiring interpretation of broader population-level genetic phenomena. While global ARI values (0.553–1.000) suggest modest overall restructuring, targeted analysis within epidemiologically relevant clusters reveals substantial internal reorganization invisible to traditional methods. In star clustering analysis, 25.8%–31.1% of *L. monocytogenes* clusters and 55.9%–60.3% of *S. enterica* clusters showed source priority reordering relative to the cgMLST baseline. Within the scope of this study, the added value of the combined cgMLST + cgDist workflow is therefore both validated—through the formally proven and empirically verified $d_{\mathrm{cgMLST}} \le d_{\mathrm{cgDist}}$ ordering invariant, confirmed for all pairwise comparisons across four datasets and two species (Supplementary Methods)—and demonstrated through these multiscale clustering and source-prioritization analyses; comprehensive benchmarking against the broader, currently nonharmonized landscape of single-method surveillance practices is a distinct question reserved for future work.

While cluster-focused analysis approaches exist in tools like PHYLOViZ and ReporTree that zoom from many samples to few samples [43, 44], cgDist provides a complementary capability: zooming from allelic distances to nucleotide-level variants within identified clusters. This deployment strategy leverages initial cgMLST screening to identify potential epidemiological clusters of interest, followed by selective application of cgDist to expand resolution from allele counts to SNP and InDel quantification within clusters requiring detailed investigation. Representative examples demonstrate systematic rather than random improvements—*Listeria* patient Lm_0880 experienced source rank changes from 4*→*8 and 7*→*4, while *Salmonella* patient Se_0003 showed a source advancing from rank 6*→*1. Such priority reorderings illustrate scenarios in which cgMLST- and cgDist-based rankings diverge for candidate sources within a cluster [3]. Whether cgDist-based ranking differences translate into distinct downstream investigative outcomes would require evaluation against ground-truth investigation records and is therefore reserved for future work.

The species-specific patterns observed in our datasets reflect the characteristic genetic features of each bacterial species: *L. monocytogenes*’ generally clonal structure yields conservative refinements within tight clusters, while *S. enterica*’ higher genetic diversity enables detection of substantial hidden variation, with some clusters fragmenting into 11 distinct genetic groups. This differential response confirms that cgDist’s value lies in targeted resolution enhancement rather than wholesale clustering restructuring.

The exploratory recombination-candidate flagging analysis enabled by the cgDist cache architecture is described in Supplementary Methods; formal validation against established recombination-detection tools is reserved for future work.

### Performance characteristics and scalability

Comprehensive benchmarking reveals that genetic diversity, assessed through the number of unique allele pairs requiring alignment computation, rather than dataset size, determines computational requirements and optimal threading strategies. High-diversity datasets achieve best performance at 16–32 threads due to memory access contention, while low-diversity datasets maintain near-linear scaling to 128 threads. The Se-1540 *Salmonella* dataset achieved 18.1*×* speedup with 112.9% efficiency at 16 threads, while the Lm-1426 *Listeria* dataset reached 125.1*×* speedup with 97.7% efficiency at 128 threads.

The unified cache architecture provides cumulative benefits for operational surveillance. Progressive cache accumulation in incremental processing validation demonstrated efficiency gains from 0% to 88.3% cache hit rates, with the 300-sample batch processing faster than the 250-sample batch due to alignment reuse. This counter-intuitive scaling behavior, where larger datasets process faster than smaller ones, validates the practical utility of persistent alignment caching in continuous surveillance workflows.

### Operational deployment and surveillance

In its operational deployment within the GENPAT bioinformatics platform, cgDist operates alongside ReporTree for cluster analysis [43] and SPREAD for interactive cluster visualization [42], thereby contributing to an end-to-end genomic surveillance workflow. Within this framework, the production Nextflow workflow achieves sub-minute execution times for cached operations.

The two-tier surveillance workflow we propose here aims to balance computational efficiency and analytical resolution through population-level screening followed by targeted cluster-zoom analysis. As an exploratory framework, its broader implementation in routine surveillance would benefit from systematic benchmarking against established single-method practices.

### Community-shared cache infrastructure

A particularly promising direction involves cgDist’s capability to generate comprehensive caches directly from cgMLST schemas without requiring actual isolate data. This enables creation of pre-computed schema-wide caches containing alignments for all possible allele pairs within a schema, which could be hosted on centralized servers and downloaded by laboratories worldwide. Such community-shared caches would eliminate initial computation barriers for new users, as laboratories could simply download pre-computed caches for their cgMLST schemas and immediately begin high-resolution surveillance without the computational overhead of building alignment data from scratch.

This transformation of cache data from institutional assets to community resources would democratize access to nucleotide-level analysis capabilities, particularly benefiting resource-constrained laboratories. The standardized nature of cgMLST schemas ensures cache compatibility across institutions, while the modular cache architecture enables selective downloading of relevant schema subsets, making this approach scalable across diverse surveillance contexts. Community-shared caches could be generated using the most common alignment parameters, ensuring broad compatibility while maintaining the flexibility for specialized applications requiring custom alignment settings.

### Comparison with alternative approaches and limitations

cgDist is positioned as a complement to standard cgMLST analysis. It operates on pre-computed cgMLST profiles, requires no reprocessing of raw sequencing data, and produces outputs compatible with the standardised interlaboratory comparability already provided by cgMLST loci. In contrast, traditional SNP-based workflows reprocess raw or assembled genome data through reference-based or reference-free variant-discovery and matrix-generation procedures, with computational requirements that may limit routine deployment at surveillance scale and may require re-running key analytical steps when new isolates are added [10–13].

A direct benchmark of cgDist against established SNP-calling pipelines (e.g. the CFSAN SNP Pipeline [11], kSNP3 [12], and related approaches reviewed in [10, 13]) would represent a natural extension of the present study. Two considerations informed the scope of this work. First, as a first-line method for cluster detection, SNP-based approaches are less practical for routine surveillance and, unlike cgMLST, still lack broadly standardized methodologies. Second, for increasing intracluster resolution, the most commonly used strategy is read mapping against a representative genome from the putative outbreak cluster; while undoubtedly informative and relevant, such analyses are better addressed in a dedicated follow-up study rather than within the scope of the present work, which establishes an enhanced, SNP-informed cgMLST framework. Accordingly, we deliberately did not perform a head-to-head comparison against a single SNP-calling workflow because no harmonized standard currently exists among bacterial SNP-calling pipelines. Different pipelines rely on different reference-selection strategies, variant-filtering criteria, and recombination-handling approaches, such that agreement (or disagreement) across pipelines remains an open question [13, 25]. Using a single pipeline as ground truth would therefore partly reflect the design choices of that specific workflow rather than independently assessing cgDist. Comprehensive benchmarking against multiple SNP-calling pipelines on shared isolate collections, including structured analysis of where and why cgDist distances diverge from SNP-pipeline outputs, represents a priority direction for future work. The present study instead focuses on algorithmic correctness (ordering invariant) and utility within cgMLST-based surveillance workflows through clustering and source-prioritization analyses across four genomic surveillance datasets.

However, several limitations warrant consideration. cgDist’s resolution remains constrained by cgMLST schema quality and comprehensiveness, though compatibility with wgMLST schemas provides enhanced discrimination for specialized applications. Optimal epidemiological thresholds established for cgMLST distance-based clustering may require recalibration for cgDist analyses, necessitating species-specific validation against known epidemiological relationships [6, 28]. More broadly, fixed allelic thresholds should be interpreted within epidemiological context, consistent with statistical, threshold-free approaches to genomic relatedness assessment [45].

Future developments should focus on expanding validation to additional bacterial species to establish species-specific epidemiological thresholds, GPU acceleration for large-scale analyses, and establishing community cache-sharing infrastructure. Extension to amino acid sequence analysis would further enable functional context for genetic variations, particularly valuable for antimicrobial resistance and virulence factor surveillance.

### Public health impact and future directions

The improved candidate–source ranking observed in our analyses may support cluster investigations by reducing the likelihood that investigative efforts focus on sources that appear close to a clinical isolate at the allele level but are more distant at the nucleotide level. The resolution gain that cgDist provides over allele-count distances applies to any cluster, including outbreak clusters; it could therefore also prove useful for outbreak investigation—a hypothesis we plan to test in future SNP-based benchmarking using epidemiologically confirmed outbreak samples as ground truth, which were not part of the present validation. We do not, however, evaluate the downstream impact of these rankings on real investigation decisions. Assessing whether nucleotide-level discrimination influences operational or regulatory outcomes would require a dedicated study using paired investigation and decision data, which is beyond the scope of the present study.

cgDist contributes to genomic epidemiology’s evolution toward real-time surveillance by bridging traditional and advanced methodologies without requiring complete system reconstruction. The standardized output formats and compatibility with established pipelines enable gradual integration while preserving institutional knowledge and analytical continuity.

As bacterial surveillance systems continue evolving toward more granular analytical capabilities, cgDist provides a methodological refinement that extends the established cgMLST framework while remaining fully compatible with it. The species-independent applicability observed across the four evaluated datasets, together with preservation of cgMLST interpretation conventions and a gradual-integration design, support the practical integration of cgDist into existing surveillance workflows. Broader benchmarking against alternative analytical pipelines and evaluation of downstream decision outcomes are reserved for future studies.

### Conclusions

cgDist addresses the balance between computational efficiency and genetic resolution in bacterial genomic surveillance by providing nucleotide-level discrimination within the operational framework of routine cgMLST workflows. The cluster-zoom paradigm defines the intended use case of cgDist: providing a precision ‘zoom lens’ for the detailed investigation of clusters identified through initial cgMLST screening. This targeted increase in resolution concentrates additional analytical effort on the clusters where it is most informative, rather than attempting to restructure global population relationships.

The algorithm’s unified cache architecture transforms genomic surveillance from batch-based processing to continuous streaming analysis, with cumulative performance benefits that improve operational efficiency over time. The improved ranking of candidate sources during cluster investigations may also support more efficient allocation of follow-up efforts; evaluation of downstream decision outcomes is reserved for future studies.

Integration into operational surveillance systems demonstrates the feasibility of deploying cgDist within production-scale infrastructure, with automated workflows achieving sub-minute execution times for cached operations. The species-independent benefits, preserved epidemiological frameworks, and gradual integration approach facilitate institutional adoption while providing a methodological foundation for continued innovation in computational genomic epidemiology.

cgDist provides a methodological refinement that adds nucleotide-level resolution within the existing cgMLST analytical framework, enabling finer discrimination between closely related isolates without the computational overhead of full SNP-calling pipelines.

## Conflict of interest

None declared.

## Supplementary Material

lqag090_Supplemental_Files

## Data Availability

All four genomic datasets used in this study are publicly available through the OHEJP BeONE Project (One Health European Joint Programme) Zenodo repositories. For each species, BeONE provides two companion deposits: a primary deposit comprising consortium-shared isolates with full source-attribution metadata, and a complementary deposit comprising additional genome assemblies selected and curated by BeONE among the Whole-Genome Sequencing data publicly available in the European Nucleotide Archive (ENA) and the NCBI Sequence Read Archive (SRA). For *Listeria monocytogenes*, the Lm-1426 dataset (1426 isolates) is available at https://doi.org/10.5281/zenodo.7802702, and the Lm-1874 dataset (1874 isolates) at https://doi.org/10.5281/zenodo.7230003. For *Salmonella enterica*, the Se-1540 dataset (1540 isolates) is available at https://doi.org/10.5281/zenodo.7802723, and the Se-1434 dataset (1434 isolates) at https://doi.org/10.5281/zenodo.7230091. ENA accession numbers for each isolate are provided in the BeONE metadata files included in those deposits. The cgMLST schemas used for analysis are the Institut Pasteur cgMLST schema (1748 loci) [8] for *Listeria monocytogenes* and the INNUENDO cgMLST schema [26] derived from the EnteroBase wgMLST schema [24] for *Salmonella enterica*, both available through ChewieNS [23] (https://chewbbaca.online/) (accessed March 2024). For *Salmonella enterica*, we applied the EFSA-filtered subset (3255 loci) following European food safety surveillance standards, available from https://doi.org/10.5281/zenodo.1323684. Cache files generated during this study are available upon request from the corresponding author due to their large file sizes. All distance matrices generated by this study (Hamming, cgDist SNPs-only, SNPs+InDel-contiguous, and SNPs+InDel-bases modes), allelic profiles, and recombination-candidate flagging outputs are provided as Supplementary Data and publicly available on Zenodo at https://doi.org/10.5281/zenodo.17285517. Complete file descriptions are available in Supplementary Data. Validation scripts are available in the cgDist repository (https://github.com/genpat-it/cgDist). The cgDist software is open-source and freely available under the MIT license. The core algorithm implementation in Rust is available at https://github.com/genpat-it/cgDist and permanently archived at Zenodo (concept DOI: https://doi.org/10.5281/zenodo.18025926, which resolves to the latest released version), with comprehensive documentation including installation instructions, usage examples, and API reference. Multi-architecture Docker images are published on every release to GitHub Container Registry (https://ghcr.io/genpat-it/cgdist).

## References

[1] Allard MW, Bell R, Ferreira CM et al. Genomics of foodborne pathogens for microbial food safety. Curr Opin Biotechnol. 2018;49:224–29. 10.1016/j.copbio.2017.11.002 29169072

[2] Didelot X, Fraser C, Gardy J et al. Genomic infectious disease epidemiology in partially sampled and ongoing outbreaks. Mol Biol Evol. 2017;34:997–1007. 28100788 10.1093/molbev/msw275 PMC5850352

[3] Pightling AW, Pettengill JB, Luo Y et al. Interpreting whole-genome sequence analyses of foodborne bacteria for regulatory applications and outbreak investigations. Frontiers Microbiol. 2018;9:1482. 10.3389/fmicb.2018.01482 PMC6048267 30042741

[4] Gymoese P, Sørensen G, Litrup E et al. Investigation of outbreaks of *Salmonella enterica serovar Typhimurium* and its monophasic variants using whole-genome sequencing, Denmark. Emerg Infect Dis. 2017;23:1631–39. 10.3201/eid2310.161248 28930002 PMC5621559

[5] Maiden MCJ, Jansen van Rensburg MJ, Bray JE et al. MLST revisited: the gene-by-gene approach to bacterial genomics. Nat Rev Microbiol. 2013;11:728–36. 10.1038/nrmicro3093 23979428 PMC3980634

[6] Ruppitsch W, Pietzka A, Prior K et al. Defining and evaluating a core genome multilocus sequence typing scheme for whole-genome sequence-based typing of *Listeria* monocytogenes. J Clin Microbiol. 2015;53:2869–76. 10.1128/JCM.01193-15 26135865 PMC4540939

[7] Silva M, Machado MP, Silva DN et al. chewBBACA: a complete suite for gene-by-gene schema creation and strain identification. Microbial Genomics. 2018;4:e000166. 10.1099/mgen.0.000166 29543149 PMC5885018

[8] Moura A, Criscuolo A, Pouseele H et al. Whole genome-based population biology and epidemiological surveillance of *Listeria* monocytogenes. Nature Microbiol. 2016;2:16185. 10.1038/nmicrobiol.2016.185 27723724 PMC8903085

[9] Carriço JA, Crochemore M, Francisco AP et al. Fast phylogenetic inference from typing data. Algorithms Mol Biol. 2018;13:4. 10.1186/s13015-017-0119-7 29467814 PMC5815242

[10] Schürch AC, Arredondo-Alonso S, Willems RJL et al. Whole genome sequencing options for bacterial strain typing and epidemiologic analysis based on single nucleotide polymorphism versus gene-by-gene-based approaches. Clin Microbiol Infect. 2018;24:350–54. 10.1016/j.cmi.2017.12.016 29309930

[11] Davis S, Pettengill JB, Luo Y et al. CFSAN SNP Pipeline: an automated method for constructing SNP matrices from next-generation sequence data. PeerJ Computer Sci. 2015;1:e20. 10.7717/peerj-cs.20

[12] Gardner SN, Slezak T, Hall BG. kSNP3.0: SNP detection and phylogenetic analysis of genomes without genome alignment or reference genome. Bioinformatics. 2015;31:2877–78. 10.1093/bioinformatics/btv271 25913206

[13] Uelze L, Grützke J, Borowiak M et al. Typing methods based on whole genome sequencing data. One Health Outlook. 2020;2:3. 10.1186/s42522-020-0010-1 33829127 PMC7993478

[14] Amdahl GM . Validity of the single processor approach to achieving large scale computing capabilities. AFIPS Conf Proc. 1967;30:483–85.

[15] Feil EJ, Holmes EC, Bessen DE et al. Recombination within natural populations of pathogenic bacteria: short-term empirical estimates and long-term phylogenetic consequences. Proc Natl Acad Sci. 2001;98:182–87. 10.1073/pnas.98.1.182 11136255 PMC14565

[16] Didelot X, Maiden MC. Impact of recombination on bacterial evolution. Trends Microbiol. 2010;18:315–22. 10.1016/j.tim.2010.04.002 20452218 PMC3985120

[17] Croucher NJ, Page AJ, Connor TR et al. Rapid phylogenetic analysis of large samples of recombinant bacterial whole genome sequences using Gubbins. Nucleic Acids Res. 2015;43:e15. 10.1093/nar/gku1196 25414349 PMC4330336

[18] Kleta S, Hammerl JA, Dieckmann R et al. Molecular tracing to find source of protracted invasive listeriosis outbreak, southern Germany, 2012-2016. Emerg Infect Dis. 2017;23:1680–83. 10.3201/eid2310.161623 28930013 PMC5621528

[19] Snitkin ES, Zelazny AM, Thomas PJ et al. Tracking a hospital outbreak of Carbapenem-resistant *Klebsiella pneumoniae* with whole-genome sequencing. Sci Transl Med. 2012;4:148ra116. 10.1126/scitranslmed.3004129 PMC3521604 22914622

[20] Matsakis ND, Klock FS The Rust language. In: Proceedings of the 2014 ACM SIGAda Annual Conference on High Integrity Language Technology (HILT 14). New York, NY, USA: ACM, 2014, 103–104. 10.1145/2663171.2663188

[21] Needleman SB, Wunsch CD. A general method applicable to the search for similarities in the amino acid sequence of two proteins. J Mol Biol. 1970;48:443–53. 10.1016/0022-2836(70)90057-4 5420325

[22] Daily J . Parasail: sIMD C library for global, semi-global, and local pairwise sequence alignments. BMC Bioinformatics. 2016;17:81. 10.1186/s12859-016-0930-z 26864881 PMC4748600

[23] Mamede R, Vila-Cerqueira P, Silva M et al. Chewie Nomenclature Server (chewie-NS): a deployable nomenclature server for easy sharing of core and whole genome MLST schemas. Nucleic Acids Res. 2021;49:D660–D666. 10.1093/nar/gkaa889 33068420 PMC7778912

[24] Alikhan N-F, Zhou Z, Sergeant MJ et al. A genomic overview of the population structure of Salmonella. PLoS Genet. 2018;14:e1007261. 10.1371/journal.pgen.1007261 29621240 PMC5886390

[25] Mixão V, Pinto M, Sobral D et al. Multi-country and intersectoral assessment of cluster congruence between pipelines for genomics surveillance of foodborne pathogens. Nat Commun. 2025;16:3961. 10.1038/s41467-025-59246-8 40295532 PMC12038046

[26] Llarena A-K, Ribeiro-Gonçalves BF, Nuno Silva D et al. INNUENDO: a cross-sectoral platform for the integration of genomics in the surveillance of food-borne pathogens. EFSA Supporting Publications. 2018;15:1498E. 10.2903/sp.efsa.2018.EN-1498

[27] European Food Safety Authority . Guidelines for Reporting Whole Genome Sequencing-based Typing Data Through the EFSA One Health WGS System. Parma, Italy: EFSA Supporting Publication EN-7413, EFSA, 2022. 10.2903/sp.efsa.2022.EN-7413

[28] Palma F, Mangone I, Janowicz A et al. *In vitro* and *in silico* parameters for precise cgMLST typing of *Listeria* monocytogenes. BMC Genomics. 2022;23:235. 10.1186/s12864-022-08437-4 35346021 PMC8961897

[29] Besser JM, Carleton HA, Trees E et al. Interpretation of whole-genome sequencing for enteric disease surveillance and outbreak investigation. Foodborne Pathogens Dis. 2019;16:504–12. 10.1089/fpd.2019.2650 PMC6653782 31246502

[30] Uelze L, Becker N, Borowiak M et al. Toward an integrated genome-based surveillance of *Salmonella enterica* in Germany. Frontiers Microbiol. 2021;12:626941. 10.3389/fmicb.2021.626941 PMC7902525 33643254

[31] Seemann T . cgmlst-dists: calculate distance matrix from ChewBBACA cgMLST allele call tables. 2020. https://github.com/tseemann/cgmlst-dists (23 April 2025, date last accessed).

[32] Pearson K . Contributions to the mathematical theory of evolution. II. Skew variation in homogeneous material. Philos Trans R Soc A. 1895;186:343–414.

[33] Spearman C . The proof and measurement of association between two things. Am J Psychol. 1904;15:72–101. 10.2307/1412159 3322052

[34] Virtanen P, Gommers R, Oliphant TE et al. SciPy 1.0: fundamental algorithms for scientific computing in Python. Nat Methods. 2020;17:261–72. 10.1038/s41592-019-0686-2 32015543 PMC7056644

[35] Hubert L, Arabie P. Comparing partitions. J Classification. 1985;2: 193–218. 10.1007/BF01908075

[36] Chen Y, Luo Y, Carleton H et al. Whole genome and core genome multilocus sequence typing and single nucleotide polymorphism analyses of Listeria monocytogenes isolates associated with an outbreak linked to cheese, United States, 2013. Appl Environ Microbiol. 2017;83:e00633–17. 10.1128/AEM.00633-17 28550058 PMC5514676

[37] Moura A, Tourdjman M, Leclercq A et al. Real-time whole-genome sequencing for surveillance of Listeria monocytogenes, France. Emerg Infect Dis. 2017;23:1462–70. 10.3201/eid2309.170336 28643628 PMC5572858

[38] Hagberg AA, Schult DA, Swart PJ. Exploring network structure, dynamics, and function using NetworkX. In: Varoquaux G, Vaught T, Millman J, (eds.), Proceedings of the 7th Python in Science Conference (SciPy 2008). Pasadena, CA USA, SciPy, 2008, pp. 11–15.

[39] Didelot X, Wilson DJ. ClonalFrameML: efficient inference of recombination in whole bacterial genomes. PLoS Comput Biol. 2015;11:e1004041. 10.1371/journal.pcbi.1004041 25675341 PMC4326465

[40] Mostowy R, Croucher NJ, Andam CP et al. Efficient inference of recent and ancestral recombination within bacterial populations. Mol Biol Evol. 2017;34:1167–82. 10.1093/molbev/msx066 28199698 PMC5400400

[41] Di Tommaso P, Chatzou M, Floden EW et al. Nextflow enables reproducible computational workflows. Nat Biotechnol. 2017;35:316–19. 10.1038/nbt.3820 28398311

[42] de Ruvo A, De Luca A, Bucciacchio A et al. SPREAD: spatiotemporal pathogen relationships and epidemiological analysis dashboard. Veterinaria Italiana. 2024;60. 10.12834/VetIt.3476.23846.1 38504601

[43] Mixão V, Pinto M, Sobral D et al. ReporTree: a surveillance-oriented tool to strengthen the linkage between pathogen genetic clusters and epidemiological data. Genome Med. 2023;15:43. 10.1186/s13073-023-01196-1 37322495 PMC10273728

[44] Nascimento M, Sousa A, Ramirez M et al. PHYLOViZ 2.0: providing scalable data integration and visualization for multiple phylogenetic inference methods. Bioinformatics. 2017;33:128–29. 10.1093/bioinformatics/btw582 27605102

[45] Radomski N, Cadel-Six S, Cherchame E et al. A simple and robust statistical method to define genetic relatedness of samples related to outbreaks at the genomic scale–application to foodborne pathogen surveillance. Frontiers Microbiol. 2019;10:2413. 10.3389/fmicb.2019.02413 PMC6821717 31708892

