# Supplementary material for: cgDist: Nucleotide-level distance calculation from cgMLST allelic profiles
Source: NAR Genom Bioinform. 2026 Sep 2;8(3):lqag090. doi: 10.1093/nargab/lqag090 (PMC13535247; doi:10.1093/nargab/lqag090)
Supplement: Supplementary file 1 [file lqag090_supplemental_files.zip › supplementary.pdf]

Supplementary Materials:
cgDist: Nucleotide-Level Distance Calculation from cgMLST Allelic Profiles

Supplementary Materials

This supplementary materials document contains three main sections: data availability, detailed methodological descriptions, and additional results. All materials are organized for clarity and ease of access.

Supplementary Data

The following supplementary data files are available with this article, organized by species for clarity and ease of access:

Listeria monocytogenes Dataset 1426

File: `Lm1426_allelic_profiles.tsv` - Allelic profile matrix (1,426 samples). Contains cgMLST profiles for all samples used in the analysis. File format: TSV.
File: `Lm1426_hamming.tsv` - Hamming distance matrix (1,426 samples). Contains pairwise Hamming distances calculated from allelic profiles using cgmlst-dists. File format: TSV.
File: `Lm1426_hamming_98percent.tsv` - Hamming distance matrix (98% sample completeness threshold). Filtered dataset containing only samples with at least 98% of loci successfully called. File format: TSV.
File: `Lm1426_cgdist_snps_indel_contiguous.tsv` - cgDist matrix - SNPs + InDel-contiguous (1,426 samples). Contains pairwise distances calculated using the cgDist algorithm counting SNPs and InDel-contiguous. File format: TSV.
File: `Lm1426_cgdist_snps_indel_contiguous_98percent.tsv` - cgDist matrix - SNPs + InDel-contiguous (98% sample completeness threshold). Filtered dataset containing only samples with at least 98% of loci successfully called. File format: TSV.
File: `Lm1426_cgdist_snps_only.tsv` - cgDist matrix - SNPs only (1,426 samples). Contains pairwise distances calculated using only SNPs. File format: TSV.
File: `Lm1426_cgdist_snps_only_98percent.tsv` - cgDist matrix - SNPs only (98% sample completeness threshold). Filtered dataset containing only samples with at least 98% of loci successfully called. File format: TSV.
File: `Lm1426_cgdist_snps_indel_bases.tsv` - cgDist matrix - SNPs + InDel bases (1,426 samples). Contains pairwise distances calculated using SNPs and total InDel bases. File format: TSV.

File: `Lm1426_cgdist_snps_indel_bases_98percent.tsv` - cgDist matrix - SNPs + InDel bases (98% sample completeness threshold). Filtered dataset containing only samples with at least 98% of loci successfully called. File format: TSV.
File: `Lm1426_flagging_per_locus_98percent.tsv` - Per-locus output of the exploratory recombination-candidate flagging analysis (see Supplementary Methods) on the Lm-1426 dataset at 98% sample completeness. The file contains per-flagged-locus records: sample pair identifiers, locus information, SNP/InDel counts, sequence lengths, and ρ values. The Lm-1426 dataset shows no flagged loci at the 3.0% ρ threshold, consistent with the predominantly clonal population structure of *Listeria monocytogenes*. File format: TSV.
File: `Lm1426_flagging_pairwise_summary_98percent.tsv` - Pairwise summary of the recombination-candidate flagging output for the Lm-1426 dataset at 98% sample completeness. Provides sample pairs with the count of loci flagged for elevated ρ, the corresponding percentage of analyzed loci, and summary statistics. File format: TSV.

Listeria monocytogenes Dataset 1874

File: `Lm1874_allelic_profiles.tsv` - Allelic profile matrix (1,874 samples). Contains cgMLST profiles for the expanded dataset. File format: TSV.
File: `Lm1874_hamming.tsv` - Hamming distance matrix (1,874 samples). File format: TSV.
File: `Lm1874_hamming_98percent.tsv` - Hamming distance matrix (98% sample completeness threshold). Filtered dataset containing only samples with at least 98% of loci successfully called. File format: TSV.
File: `Lm1874_cgdist_snps_indel_contiguous.tsv` - cgDist matrix - SNPs + InDel-contiguous (1,874 samples). File format: TSV.
File: `Lm1874_cgdist_snps_indel_contiguous_98percent.tsv` - cgDist matrix - SNPs + InDel-contiguous (98% sample completeness threshold). Filtered dataset containing only samples with at least 98% of loci successfully called. File format: TSV.
File: `Lm1874_cgdist_snps_only.tsv` - cgDist matrix - SNPs only (1,874 samples). File format: TSV.
File: `Lm1874_cgdist_snps_only_98percent.tsv` - cgDist matrix - SNPs only (98% sample completeness threshold). Filtered dataset containing only samples with at least 98% of loci successfully called. File format: TSV.
File: `Lm1874_cgdist_snps_indel_bases.tsv` - cgDist matrix - SNPs + InDel bases (1,874 samples). File format: TSV.
File: `Lm1874_cgdist_snps_indel_bases_98percent.tsv` - cgDist matrix - SNPs + InDel bases (98% sample completeness threshold). Filtered dataset containing only samples with at least 98% of loci successfully called. File format: TSV.
File: `Lm1874_flagging_per_locus_98percent.tsv` - Per-locus output of the exploratory recombination-candidate flagging analysis (see Supplementary Methods) on the extended Lm-1874 dataset at 98% sample completeness. The file contains per-flagged-locus records using the same schema as the Lm-1426 file. The Lm-1874 dataset likewise shows no flagged loci at the 3.0% ρ threshold. File format: TSV.
File: `Lm1874_flagging_pairwise_summary_98percent.tsv` - Pairwise summary of the recombination-candidate flagging output for the Lm-1874 dataset at 98% sample completeness. Same schema as the Lm-1426 pairwise summary file. File format: TSV.

Salmonella enterica Dataset 1540

File: `Se1540_allelic_profiles.tsv` - Allelic profile matrix (1,540 samples). Contains cgMLST profiles for all samples used in the analysis. File format: TSV.
File: `Se1540_hamming.tsv` - Hamming distance matrix (1,540 samples). Contains pairwise Hamming distances calculated from allelic profiles. File format: TSV.
File: `Se1540_hamming_98percent.tsv` - Hamming distance matrix (98% sample completeness threshold). Filtered dataset containing only samples with at least 98% of loci successfully called. File format: TSV.
File: `Se1540_cgdist_snps_indel_contiguous.tsv` - cgDist matrix - SNPs + InDel-contiguous (1,540 samples). Contains pairwise distances calculated using the cgDist algorithm. File format: TSV.
File: `Se1540_cgdist_snps_indel_contiguous_98percent.tsv` - cgDist matrix - SNPs + InDel-contiguous (98% sample completeness threshold). Filtered dataset containing only samples with at least 98% of loci successfully called. File format: TSV.
File: `Se1540_cgdist_snps_only.tsv` - cgDist matrix - SNPs only (1,540 samples). Contains pairwise distances calculated using only SNPs. File format: TSV.
File: `Se1540_cgdist_snps_only_98percent.tsv` - cgDist matrix - SNPs only (98% sample completeness threshold). Filtered dataset containing only samples with at least 98% of loci successfully called. File format: TSV.
File: `Se1540_cgdist_snps_indel_bases.tsv` - cgDist matrix - SNPs + InDel bases (1,540 samples). Contains pairwise distances calculated using SNPs and total InDel bases. File format: TSV.
File: `Se1540_cgdist_snps_indel_bases_98percent.tsv` - cgDist matrix - SNPs + InDel bases (98% sample completeness threshold). Filtered dataset containing only samples with at least 98% of loci successfully called. File format: TSV.
File: `Se1540_flagging_per_locus_98percent.tsv` - Per-locus output of the exploratory recombination-candidate flagging analysis (see Supplementary Methods) on the Se-1540 dataset at 98% sample completeness. At the 3.0% ρ threshold, the Se-1540 dataset has 20 flagged sample pairs (0.03% of EFSA loci), reflecting the greater diversity observed in the Se-1540 dataset relative to the *Listeria* datasets; downstream interpretation of these candidate regions would require dedicated tools (Gubbins, ClonalFrameML, fastGEAR; see Supplementary Methods). The file contains per-flagged-locus records: sample pair identifiers, locus information, SNP/InDel counts, sequence lengths, and ρ values. File format: TSV.
File: `Se1540_flagging_pairwise_summary_98percent.tsv` - Pairwise summary of the recombination-candidate flagging output for the Se-1540 dataset at 98% sample completeness. Provides sample pairs with the count of loci flagged for elevated ρ, the corresponding percentage of analyzed loci, and summary statistics. File format: TSV.
File: `Se1540_rho_distribution.png` - Visualization of the empirical ρ (within-allele nucleotide-difference density; see Supplementary Methods) in the Se-1540 dataset, used to inform the selection of the 3.0% candidate-flagging threshold. Includes histogram, cumulative distribution, threshold ranges analysis, and log-scale distribution plots. File format: PNG.

Salmonella enterica Dataset 1434

File: `Se1434_allelic_profiles.tsv` - Allelic profile matrix (1,434 samples). Contains cgMLST profiles for the validation dataset. File format: TSV.

File: `Se1434_hamming.tsv` - Hamming distance matrix (1,434 samples). File format: TSV.
File: `Se1434_hamming_98percent.tsv` - Hamming distance matrix (98% sample completeness threshold). Filtered dataset containing only samples with at least 98% of loci successfully called. File format: TSV.
File: `Se1434_cgdist_snps_indel_contiguous.tsv` - cgDist matrix - SNPs + InDel-contiguous (1,434 samples). File format: TSV.
File: `Se1434_cgdist_snps_indel_contiguous_98percent.tsv` - cgDist matrix - SNPs + InDel-contiguous (98% sample completeness threshold). Filtered dataset containing only samples with at least 98% of loci successfully called. File format: TSV.
File: `Se1434_cgdist_snps_only.tsv` - cgDist matrix - SNPs only (1,434 samples). File format: TSV.
File: `Se1434_cgdist_snps_only_98percent.tsv` - cgDist matrix - SNPs only (98% sample completeness threshold). Filtered dataset containing only samples with at least 98% of loci successfully called. File format: TSV.
File: `Se1434_cgdist_snps_indel_bases.tsv` - cgDist matrix - SNPs + InDel bases (1,434 samples). File format: TSV.
File: `Se1434_cgdist_snps_indel_bases_98percent.tsv` - cgDist matrix - SNPs + InDel bases (98% sample completeness threshold). Filtered dataset containing only samples with at least 98% of loci successfully called. File format: TSV.
File: `Se1434_flagging_per_locus_98percent.tsv` - Per-locus output of the exploratory recombination-candidate flagging analysis (see Supplementary Methods) on the validation Se-1434 dataset at 98% sample completeness. At the 3.0% ρ threshold, the Se-1434 dataset shows minimal flagging activity (one sample pair). The file contains per-flagged-locus records using the same schema as the Se-1540 file. File format: TSV.
File: `Se1434_flagging_pairwise_summary_98percent.tsv` - Pairwise summary of the recombination-candidate flagging output for the Se-1434 dataset at 98% sample completeness. Same schema as the Se-1540 pairwise summary file. File format: TSV.

Summary

All supplementary data files are provided in Tab-Separated Values (TSV) format for maximum compatibility with computational biology software. Distance matrices include metadata headers with analysis parameters and timestamps for reproducibility. The complete dataset enables researchers to reproduce all analyses presented in this study and conduct their own comparative studies.

Files are organized by species and dataset size with consistent naming conventions. The allelic profiles provide the raw cgMLST data serving as input for all distance calculations, while Hamming matrices contain traditional allelic difference calculations establishing baseline comparisons. The cgDist matrices encompass three calculation modes: SNPs+InDel-contiguous counting both SNPs and maximal contiguous indel regions in the alignment, SNPs only focusing exclusively on single nucleotide changes, and SNPs+InDel bases weighting InDels by total affected nucleotides. Data files use the suffix `snps_indel_contiguous` matching the current CLI mode flag. The legacy mode flag `snps-indel-events` remains accepted by the cgDist CLI as a deprecated alias for backward compatibility. Files designated with the 98% threshold suffix represent high-quality subsets filtered for samples with at least 98% of loci successfully called. The Se-1540 dataset additionally includes outputs from the exploratory recombination-candidate flagging analysis (Section S6), to-

gether with the empirical ρ distribution used to inform selection of the 3.0% flagging threshold.

The dataset supports reproducible research and enables the scientific community to evaluate cgDist performance across multiple pathogen species and analytical scenarios. Each species section contains analytical workflows from raw allelic profiles through all distance calculation modes, enabling researchers to reproduce analyses or adapt methods to their own surveillance datasets. The Se-1540 dataset additionally includes outputs from the exploratory recombination-candidate flagging analysis, including per-pair output and the empirical ρ distribution used to inform threshold selection. The results are presented as a feasibility demonstration of the flagging approach rather than as validated recombination-detection results (Section S6).

Supplementary Methods

S1 Debugging and Quality Assurance Capabilities

While cgDist's standard operation focuses on efficient aggregate distance metrics, the tool architecture supports detailed debugging and quality assurance through optional preservation of alignment details. Users can optionally enable verbose logging and detailed alignment output that captures the complete Parasail alignment results, including alignment matrices, trace-back paths, and variant positions. This debugging capability enables researchers to manually inspect the specific nucleotide differences contributing to distance calculations, validate alignment quality, and identify potential sequencing or assembly artifacts that might affect epidemiological interpretation.

The debugging features encompass complete alignment matrices showing the dynamic programming calculations, trace-back paths indicating the best alignment route through the scoring matrix, detailed variant positions with nucleotide-level annotations for each detected difference, per-locus alignment statistics and quality metrics enabling identification of problematic regions, and comprehensive validation reports for cache integrity and consistency verification.

However, enabling comprehensive alignment debugging significantly increases storage requirements and computational overhead, making it suitable primarily for validation studies, method development, or troubleshooting specific analytical scenarios rather than routine surveillance applications. For routine surveillance, the standard mode provides sufficient information through summary statistics while maintaining good performance.

S2 Detailed Distance Calculation Algorithm

The following algorithm provides the complete technical details for analyzing pairwise sequence alignments and computing different distance metrics from the classified variants.

Algorithm 1: Distance Calculation from Sequence Alignment

```
Input: Two sequences seq_1, seq_2, distance mode M
Output: Distance value d
// Step 1: Perform global sequence alignment
alignment ← GlobalAlign(seq_1, seq_2) using Parasail library;
(aligned_1, aligned_2) ← Extract aligned sequences from alignment;
// Step 2: Initialize counters
snps ← 0, indel_contiguous ← 0, indel_bases ← 0;
in_gap ← false, gap_type ← null;
// Step 3: Analyze alignment character by character
for i ← 0 to length(aligned_1) - 1 do
    c_1 ← aligned_1[i], c_2 ← aligned_2[i];
    if c_1 = c_2 then
        // Identical nucleotides: end any gap
        in_gap ← false;
    else if c_1 ≠ '-' and c_2 ≠ '-' then
        // Mismatch: SNP detected
        snps ← snps + 1;
        in_gap ← false;
    else
        // Gap detected: insertion or deletion
        if not in_gap then
            // Start of new InDel-contiguous
            indel_contiguous ← indel_contiguous + 1;
            in_gap ← true;
            gap_type ← (c_1 = '-' ? "insertion" : "deletion");
        indel_bases ← indel_bases + 1;
// Step 4: Apply distance mode (with optional Hamming fallback in SNPs-only mode)
switch M do
    case SNPs-only do
        if snps > 0 then
            d ← snps;
        else if hamming_fallback_enabled and indel_contiguous > 0 then
            d ← 1 ;                      // Optional Hamming fallback for InDel-only differences
        else
            d ← 0;
    case SNPs + InDel-contiguous do
        d ← snps + indel_contiguous;
    case SNPs + InDel bases do
        d ← snps + indel_bases;
    case Hamming do
        d ← 1 ;                                              // Single allelic difference
return d;
```

S3 Alignment Parameters and Technical Details

Alignment Parameter Settings

cgDist employs the DNA-strict alignment mode with a match score of +3, mismatch penalty of -2, gap opening penalty of 8, and gap extension penalty of 3.

This predefined parameter set is optimized for bacterial genomic sequences, with elevated gap penalties designed to favor SNP-based explanations over complex InDel patterns while minimizing alignment artifacts. The conservative parameter selection enhances discriminatory power within epidemiologically relevant distance ranges important for cluster characterization.

Implementation Details

The cgDist algorithm is implemented in Rust 2021 edition for memory safety and performance, utilizing Parasail-rs v0.3.5 Rust bindings for sequence alignment with SIMD vectorization support. The system employs native Rust threading with a work-stealing scheduler for parallelization, LZ4 algorithm for efficient cache compression, and compressed JSON format with metadata headers for cache storage. Supported hashers include CRC32 (default), MD5, SHA256, sequence-based, and Hamming algorithms.

The Parasail library provides optimized C implementation with Rust bindings, ensuring both computational efficiency and memory safety while maintaining deterministic alignment results required for reproducible distance calculations.

S4 Detailed Computational Complexity Analysis

To analyze cgDist computational requirements, we perform formal complexity analysis examining time complexity $T(n, s, l)$ and space complexity $S(n, u, l)$, where n represents the number of genomic samples, s the maximum nucleotide sequence length per locus in the schema, l the number of cgMLST loci, and u the average number of unique alleles per locus in the schema.

Time Complexity Analysis

Upper Bound: The algorithm computes pairwise distances for $\binom{n}{2} = \frac{n(n-1)}{2} \in O(n^2)$ unique sample pairs. For each pair (i, j), we compare l cgMLST loci, performing sequence alignment when alleles differ. The alignment cost per locus is $O(s^2)$ for the worst-case maximum sequence length using the Needleman-Wunsch algorithm. Therefore:

$$T(n, s, l) = \sum_{i=1}^{n-1} \sum_{j=i+1}^{n} \sum_{\ell=1}^{l} O(s^2) = O(n^2) \times l \times O(s^2) = O(n^2 \times s^2 \times l)$$

Lower Bounds: The algorithm must examine each sample pair at least once ($\Omega(n^2)$) and process all loci for meaningful distances ($\Omega(l)$), establishing $T(n, s, l) \in \Omega(n^2 \times l)$. In the worst case where all alleles differ, sequence alignment adds $\Omega(s^2)$ per comparison, yielding the tight bound $T(n, s, l) \in \Theta(n^2 \times l \times s^2)$.

Optimization: Cache optimization reduces effective time complexity to $O(n^2 \times l)$ with high hit rates, transforming $O(s^2)$ alignment operations into $O(1)$ lookups. Parallelization achieves $T_{\text{parallel}} = O(n^2 \times s^2 \times l/p)$ for p processors, limited by Amdahl's law and cache contention.

Space Complexity Analysis

Memory Requirements: Memory requirements include the symmetric distance matrix $O(n^2)$, alignment cache storage $O(l \times u^2)$ for allele pair alignments, allelic profiles $O(n \times l)$, and temporary alignment buffers $O(s^2)$ for dynamic programming matrices (allocated per alignment operation). The dominant term depends on dataset characteristics:

$$S(n, u, l) = O(n^2) + O(l \times u^2) + O(n \times l) + O(s^2) = O(n^2 + l \times u^2)$$

Dominance Cases: For small datasets with high allelic diversity ($n^2 < l \times u^2$), cache storage dominates memory usage. For large sample collections with moderate diversity ($n^2 > l \times u^2$), the distance matrix becomes the primary memory constraint.

Practical Performance Considerations

The actual performance of cgDist depends heavily on several factors including cache hit rates, genetic diversity, sequence length distribution, and parallelization efficiency. In bacterial surveillance with clonal population structures, many allele pairs are shared across samples, leading to high cache utilization. Highly diverse populations require more unique alignments but benefit more from resolution enhancement, while shorter sequences reduce alignment costs while maintaining epidemiological utility. Parallelization efficiency depends on work distribution across processors with minimal cache contention.

S5 Mathematical Foundation: cgMLST-cgDist Ordering Theorem

We formalized the fundamental relationship between cgMLST allele-count distances and nucleotide-level distances calculated through the following ordering theorem.

Theorem 1 (cgMLST-cgDist Ordering, conditional)**.** *Let S_i and S_j be two genomic samples analyzed using the same cgMLST scheme. Assume cgDist is executed either (i) in a mode including InDel contributions (SNPs+InDel-contiguous, SNPs+InDel-bases, or Hamming), or (ii) in SNPs-only mode with the optional Hamming fallback enabled. Under either configuration,*

$$d_{cgMLST}(S_i, S_j) \leq d_{cgDist}(S_i, S_j).$$

The inequality is not guaranteed when cgDist is run in SNPs-only mode with the Hamming fallback disabled, since allele pairs differing only by InDels then contribute 0 *to the cgDist distance while contributing* 1 *to the cgMLST allele-count distance.*

To prove this theorem, we first establish the following supporting lemma:

Lemma 2. *For any locus ℓ at which the two samples have different cgMLST allele identifiers ($a_\ell^{(i)} \neq a_\ell^{(j)}$), the corresponding per-locus nucleotide-level contribution computed by cgDist satisfies $\mu(a_\ell^{(i)}, a_\ell^{(j)}) \geq 1$.*

Proof of Lemma 2. Since alleles $a_\ell^{(i)}$ and $a_\ell^{(j)}$ have different identifiers, their underlying DNA sequences must differ at least at one nucleotide position. This difference may appear as: (1) one or more single nucleotide polymorphisms (SNPs), (2) one or more insertions or deletions (InDels), or (3) a combination of both. In all cases, $\mu(a_\ell^{(i)}, a_\ell^{(j)}) \geq 1$.

In the special case of SNPs-only mode where sequence alignment reveals zero SNPs despite differing allele identifiers (indicating InDel-only variation), the optional Hamming fallback, when enabled, assigns $\mu(a_\ell^{(i)}, a_\ell^{(j)}) = 1$ to preserve the inequality. When the Hamming fallback is disabled, $\mu(a_\ell^{(i)}, a_\ell^{(j)}) = 0$ at such loci, and the inequality may therefore be violated. The distinction between these two operating regimes is documented in the main text. The remainder of this proof assumes the configuration in which the inequality holds (i.e., a mode including InDel contributions, or SNPs-only mode with the Hamming fallback enabled). ∎

Proof of Theorem 1. Let $S_i = (a_1^{(i)}, a_2^{(i)}, \ldots, a_k^{(i)})$ and $S_j = (a_1^{(j)}, a_2^{(j)}, \ldots, a_k^{(j)})$ be two allelic profiles across k cgMLST loci. Define $D = \{\ell : a_\ell^{(i)} \neq a_\ell^{(j)}\}$ as the set of loci with differing allele identifiers.

By definition, the cgMLST allele-count distance is: $d_{\text{cgMLST}}(S_i, S_j) = |D|$.

The cgDist distance is computed as: $d_{\text{cgDist}}(S_i, S_j) = \sum_{\ell=1}^{k} \mu(a_\ell^{(i)}, a_\ell^{(j)})$, where $\mu(a_\ell^{(i)}, a_\ell^{(j)})$ denotes the nucleotide-level contribution at locus ℓ.

For loci where the alleles are identical ($\ell \notin D$), we have $\mu(a_\ell^{(i)}, a_\ell^{(j)}) = 0$.

Therefore:

$$
\begin{aligned}
d_{\text{cgDist}}(S_i, S_j) &= \sum_{\ell=1}^{k} \mu(a_\ell^{(i)}, a_\ell^{(j)}) && (1)\\
&= \sum_{\ell \in D} \mu(a_\ell^{(i)}, a_\ell^{(j)}) && (2)\\
&\geq \sum_{\ell \in D} 1 \quad \text{(by Lemma 2)} && (3)\\
&= |D| && (4)\\
&= d_{\text{cgMLST}}(S_i, S_j) && (5)
\end{aligned}
$$

■

Under the configuration in which the theorem applies (i.e., modes with InDel contributions, or SNPs-only mode with Hamming fallback enabled), cgDist provides a conservative refinement of the traditional cgMLST distance: it never produces a smaller value than the corresponding cgMLST distance while exposing within-allele nucleotide variation when sequence alignment succeeds.

In SNPs-only mode with the Hamming fallback disabled, however, the cgDist distance may become strictly smaller than the cgMLST distance for allele pairs differing exclusively by InDels. This behaviour is intentional and reflects the explicit user choice to count only nucleotide-substitution events.

Corollary 3. *In SNPs-only mode with the Hamming fallback enabled, equality $d_{cgMLST}(S_i, S_j) = d_{cgDist}(S_i, S_j)$ holds when every differing allele pair either: (1) contains exactly one SNP (regardless of the presence of InDels), or (2) contains zero SNPs and therefore triggers the fallback contribution.*

S6 Detailed Methods for the Recombination-Candidate Flagging Companion Analysis

Recombination-Candidate Flagging Framework

This section describes an exploratory companion analysis enabled by the cgDist cache architecture. We emphasize at the outset that the procedure presented here is a heuristic recombination-candidate flagging approach rather than a validated recombination-detection method. Loci flagged by elevated within-allele nucleotide-difference density may correspond to horizontal gene transfer, but they may also arise from accumulated single-nucleotide substitutions, hypermutator phenotypes, or schema-design choices involving intrinsically diverse loci. Confirmation of recombination at flagged regions would require dedicated tools such as Gubbins [1], ClonalFrameML [2], or fastGEAR [3], and is reserved for future work.

When cgDist caches are enriched with nucleotide sequence-length information from the schema using the `--enrich-lengths` parameter, the system can compute per-locus

nucleotide-difference densities without additional computational overhead. The enrichment stores the original sequence lengths alongside alignment statistics in the cache, enabling density calculations ($\rho = \frac{\text{nucleotide differences}}{\text{average allele length}}$) in subsequent analyses. The flagging step uses elevated ρ as a heuristic indicator only: loci for which the nucleotide-difference count is high relative to allele length are flagged for follow-up examination.

Algorithmic Implementation

The recombination-candidate flagging analysis applies three key filtering steps: epidemiological filtering, ρ computation, and locus-level filtering. Epidemiological filtering restricts the analysis to sample pairs within a specified Hamming distance threshold ($\leq$15 alleles in the present datasets), therefore focusing on epidemiologically plausible comparisons. The ρ computation uses $\rho = \frac{\text{SNPs+InDel-contiguous count}}{(\text{length}_1+\text{length}_2)/2} \times 100\%$ for each allele pair. Allele pairs with ρ values exceeding the selected threshold (3.0% for Se-1540) are flagged as candidate regions for follow-up examination. Locus-level filtering can additionally be restricted to optional locus subsets (e.g., EFSA core loci for *Salmonella enterica*).

Algorithm Workflow

The algorithm processes enriched cache entries through a systematic workflow that begins by loading enriched cache containing alignment statistics and sequence lengths, then parsing allelic profiles to map CRC hashes to samples. The system calculates Hamming distances between sample pairs and filters pairs by epidemiological relevance using the Hamming threshold. For each qualifying sample pair, the algorithm analyzes all shared loci by computing the within-allele nucleotide-difference density ρ for each allele pair, comparing the resulting value ρ against the selected flagging threshold, and recording loci that exceed it. The system generates both a per-locus flagging report and a pairwise summary.

Output Files and Interpretation

The recombination-candidate flagging analysis generates two complementary output files. We emphasize that these outputs represent heuristic flagging results rather than validated recombination-detection results (see disclaimer at the beginning of this section).

Per-locus flagging file (`{Dataset}_flagging_per_locus_98percent.tsv`) contains one record per flagged allele pair for samples meeting 98% completeness. Includes sample pair identifiers, locus information, SNP counts, InDel counts, sequence lengths, and the computed ρ value, along with allele CRC identifiers for traceability.

Pairwise summary file (`{Dataset}_flagging_pairwise_summary_98percent.tsv`) provides, for each sample pair in the 98% completeness subset, the total count of loci flagged for elevated ρ, the corresponding percentage of analyzed loci, and summary statistics.

Practical Implementation

Cache enrichment is performed during the initial analysis by adding the `--enrich-lengths` flag to standard cgDist execution. Once enriched, the cache contains both alignment statistics and sequence length data, enabling recombination analysis in subsequent runs

using the `--candidate-recombination-log` parameter without additional computational overhead.

This design allows laboratories to enable the recombination-candidate flagging companion analysis retroactively on existing analyses by enriching their established cache files. The modular approach allows the flagging step to be added to existing workflows without disrupting established analytical pipelines.

Intended Use

The recombination-candidate flagging companion analysis is intended to support cluster-investigation workflows by highlighting loci whose within-allele nucleotide-difference density (ρ) is unusually elevated relative to the rest of the schema in the analyzed dataset. Such loci represent heuristic candidates that may warrant follow-up examination with dedicated horizontal-gene-transfer detection tools (e.g., Gubbins, ClonalFrameML, fastGEAR; see Supplementary Methods). cgDist itself does not classify the underlying evolutionary mechanism, distinguish recombination from accumulated single-nucleotide substitutions or hypermutator phenotypes, or infer gene-flow patterns. The flagging output may nevertheless be useful for selecting subsets of loci or sample pairs for downstream phylogenetic examination, but it should not be interpreted as a primary recombination-detection step or as evidence of horizontal gene transfer in any individual comparison.

Recombination-Candidate Flagging Algorithm

The following pseudocode describes the recombination-candidate flagging algorithm implemented in cgDist:

Algorithm 2: Recombination-Candidate Flagging from Enriched Cache

```
Input: Enriched cache C, allelic profiles P, loci filter L, density threshold τ, Hamming threshold H
Output: Per-locus flagging file (flagging_per_locus), pairwise summary file (flagging_pairwise_summary)
// Step 1: Load and validate inputs
Load enriched cache C with alignment statistics and sequence lengths;
Load allelic profiles P mapping samples to loci;
Load loci filter L (e.g., EFSA core loci) or use all loci;
// Step 2: Build sample-to-CRC mapping
sample_crcs ← ∅;
for each sample s in P do
    sample_crcs[s] ← {(locus, crc) : (locus, allele) ∈ P[s], crc = hash(allele)};
// Step 3: Calculate Hamming distances and filter pairs
valid_pairs ← ∅;
for each sample pair (s_i, s_j) where i < j do
    hamming_dist ← CountAllelicDifferences(P[s_i], P[s_j]);
    if hamming_dist ≤ H then
        valid_pairs ← valid_pairs ∪ {(s_i, s_j)};
// Step 4: Analyze candidate flagging for each valid pair
flagged_events ← ∅;
pairwise_summary ← ∅;
for each pair (s_i, s_j) ∈ valid_pairs in parallel do
    events ← ∅;
    total_loci ← 0;
    flagged_loci ← 0;
    for each locus ℓ ∈ L do
        if ℓ present in both P[s_i] and P[s_j] then
            total_loci ← total_loci + 1;
            crc_1 ← hash(P[s_i][ℓ]), crc_2 ← hash(P[s_j][ℓ]);
            if crc_1 ≠ crc_2 then
                // Different alleles: compute candidate-flagging density
                stats ← C[(ℓ, crc_1, crc_2)] ;                    // Get cached alignment stats
                length_1 ← C.get_length(crc_1), length_2 ← C.get_length(crc_2);
                avg_length ← (length_1 + length_2)/2;
                total_nuc_diffs ← stats.snps + stats.indel_contiguous;
                ρ ← (total_nuc_diffs/avg_length) × 100;
                if ρ > τ then
                    flagged_loci ← flagged_loci + 1;
                    event ← CreateFlaggingRecord(s_i, s_j, ℓ, stats, ρ);
                    events ← events ∪ {event};
    // Store results
    flagged_events ← flagged_events ∪ events;
    percentage ← (flagged_loci/total_loci) × 100;
    summary ← CreateSummary(s_i, s_j, total_loci, flagged_loci, percentage);
    pairwise_summary ← pairwise_summary ∪ {summary};
// Step 5: Generate output files
WriteDetailedEvents(flagged_events, "{Dataset}_flagging_per_locus_98percent.tsv");
WritePairwiseSummary(pairwise_summary, "{Dataset}_flagging_pairwise_summary_98percent.tsv");
return Output file paths;
```

Supplementary Results

This section will contain additional results, extended analyses, and detailed performance comparisons that support the main findings presented in the manuscript.

S7 Scalability and Performance Analysis

We conducted comprehensive scalability analysis using high-performance computing infrastructure to evaluate cgDist performance characteristics across different dataset sizes and genetic diversity levels. Testing was performed on a 128-core server with 720 GB RAM running AlmaLinux 8.10, including thread scaling from 1 to 128 cores, cache performance measurement across multiple runs, and comparison with existing tools.

Parallelization Performance and Dataset-Dependent Scalability Patterns

cgDist demonstrates dataset-dependent scalability patterns that correlate strongly with genetic diversity rather than dataset size. Comprehensive scalability testing revealed two distinct performance profiles reflecting the underlying biological characteristics of the analyzed populations.

For the high-diversity Se-1540 *Salmonella enterica* dataset (6.05M unique pairs, genetic diversity index 0.037), best performance occurs at 16-32 threads with diminishing returns beyond this range. The best configuration at 16 threads delivers 18.1× speedup with 112.9% efficiency, achieving peak throughput of 2,077 pairs/second. While maximum throughput of 3,939 pairs/second is achieved at 64 threads, efficiency drops substantially to 53.5%. Beyond 64 threads, severe thread contention results in performance collapse, with 128 threads showing only 0.4% efficiency. Memory requirements remain stable at 3.5 GB across all thread configurations, indicating that memory usage is independent of parallelization strategy.

In contrast, the low-diversity Lm-1426 *Listeria monocytogenes* dataset (0.72M unique pairs, genetic diversity index 0.018) exhibits near-linear scaling across the entire thread range. The system achieves 125.1× speedup at 128 threads with 97.7% efficiency, maintaining consistent per-thread performance of approximately 42 pairs/second across all configurations. Peak performance reaches 5,378 pairs/second at maximum thread utilization without performance degradation. Memory efficiency is notable, requiring only 1.9 GB (45% reduction compared to high-diversity datasets), demonstrating that genetic diversity rather than dataset size determines memory requirements.

Unified Distance Cache Architecture Performance

cgDist's unified distance cache system provides substantial performance improvements for multi-mode analysis workflows, particularly important for surveillance applications requiring multiple distance calculation modes. Cache performance evaluation across both species using production datasets demonstrates the significant efficiency gains achievable through intelligent caching strategies.

The cache architecture exhibits notable performance differentials between cache-building and cache-utilization phases. For the *Salmonella enterica* Se-1540 dataset (6.05M pairs), initial SNP analysis with cache building processes 2,007 pairs/second requiring 50:15 execution time and 5.57 GB memory. However, subsequent SNPs+indel-contiguous analysis

using cached alignments achieves 28,374 pairs/second (14× improvement) with significantly reduced execution time of 3:33 and 29% memory reduction to 3.95 GB. Similarly, SNPs+indel-bases analysis maintains 20,938 pairs/second processing rate with comparable memory efficiency.

For the *Listeria monocytogenes* Lm-1874 dataset (4.26M pairs), cached SNP analysis achieves 25,900 pairs/second completing in 2:44 with 3.18 GB memory usage. When building cache for SNPs+indel-contiguous analysis, processing rate drops to 1,617 pairs/second requiring 43:54 execution time and 4.51 GB memory. Subsequent cached SNPs+indel-bases analysis recovers to 20,470 pairs/second with reduced memory requirements, demonstrating 94% time reduction when reusing caches across analysis modes.

Threading Performance Analysis

Performance scaling was evaluated across thread counts from 1 to 128 cores using representative datasets from each species. The analysis measured alignment throughput as unique allele pairs processed per second, memory utilization including peak and average memory consumption patterns, cache efficiency through hit rates and lookup performance across thread counts, and load balancing via work distribution across available processors.

Comparative Performance Benchmarking

cgDist performance was compared against `cgmlst-dists` [4] for baseline cgMLST allele-count distance computation. Benchmarks included computation time as wall-clock time for distance matrix generation, memory requirements measured as peak memory usage across dataset sizes, and scalability across dataset sizes.

S8 Incremental Processing Validation

To demonstrate practical deployment capability for real-time surveillance, we developed a Nextflow pipeline for incremental processing validation. This pipeline simulates the temporal arrival of isolates in surveillance scenarios and measures the computational performance, cache efficiency, and cumulative benefits of alignment reuse as surveillance programs continuously integrate new genomic data. This workflow is integrated with the GENPAT genomic surveillance platform rather than packaged as a standalone application; we therefore describe the validation procedure in full below rather than releasing platform-specific pipeline code, while the underlying cgDist component that performs the cache-aware distance computation remains openly available (see Code Availability).

Nextflow Pipeline Architecture

The incremental processing pipeline implements a systematic workflow that establishes initial cache by processing the first dataset to build baseline cache, performs incremental updates by sequentially adding new isolates simulating real-time arrival, conducts performance monitoring to track computation time, memory usage, and cache statistics, executes validation checks to ensure distance matrix consistency across incremental updates, and measures efficiency metrics including cache hit rates and alignment reuse benefits.

Experimental Design

The validation approach enables assessment of incremental processing performance and cache efficiency benefits in realistic surveillance deployment scenarios. The pipeline processes datasets in temporal simulation mode, measuring cache growth patterns to determine how cache size and hit rates evolve with dataset growth, computational efficiency through reduction in processing time for new isolates, memory scaling as surveillance datasets expand, and update latency representing the time required to integrate new isolates into existing analyses.

Production Surveillance Integration Results

Incremental processing validation confirms that cgDist's cache-aware design enables practical deployment of nucleotide-level genomic surveillance at the scale and speed required for modern public health practice. Cache hit rates improve progressively from initial runs to subsequent analyses as surveillance programs accumulate alignment data over time, demonstrating the cumulative performance benefits needed for continuous pathogen monitoring workflows.

The practical utility of cgDist extends beyond algorithmic improvements to encompass comprehensive integration into real-world genomic surveillance workflows. cgDist has been successfully integrated into operational surveillance systems and provides a foundation for continuous pathogen monitoring in public health laboratories.

cgDist has been successfully integrated into the GENPAT bioinformatics platform, where it serves as the core distance calculation engine for real-time epidemiological analysis. The integration demonstrates production-ready capabilities including sub-minute execution times for cached operations, efficient incremental updates as new samples are added to surveillance databases, and 94% time reduction when reusing caches across different distance calculation modes.

Surveillance Performance Validation Results

Validation using incremental *Salmonella enterica* batches demonstrates cache performance improvements for real-time genomic surveillance deployment (Supplementary Table 1). The test configuration utilized the cgdist-resources schema with EFSA loci filtering (3,255 loci) in SNPs+InDel-contiguous mode with DNA-strict alignment parameters.

Progressive cache accumulation reveals efficiency gains: initial processing of 50 samples required 59.8 seconds with 0% cache efficiency, establishing the baseline cache of 267,231 alignments. The first incremental update to 100 samples achieved immediate performance improvement with 56.5% cache hit rate, reducing processing time to 56.6 seconds despite doubling the sample size. Subsequent batches demonstrated accelerating efficiency gains: 150 samples processed in 38.4 seconds (79.3% cache hit), 200 samples in 82.2 seconds (66.4% cache hit), and 250 samples in 108.9 seconds (68.6% cache hit).

The 300-sample batch completed in 61.3 seconds despite being the largest dataset—faster than the 250-sample batch—due to achieving 88.3% cache hit rate. This counterintuitive result, where larger datasets process faster than smaller ones, demonstrates the impact of cache-aware architecture on surveillance scalability. The cache grew progressively from 8.9MB (267K alignments) to 49.4MB (1.48M alignments), maintaining compression efficiency through LZ4 encoding with approximately 33 bytes per alignment entry, providing a reusable computational resource for ongoing surveillance operations.

Supplementary Table 1: Progressive cache performance improvements in cgDist surveillance pipeline validation. Incremental batches of *Salmonella enterica* samples demonstrate cache efficiency gains from 0% to 88.3% over six surveillance updates, with the 300-sample batch processing faster than the 250-sample batch due to improved cache utilization.

Batch	Samples	Time (s)	Cache Hit (%)	Efficiency (%)	Cache Entries	Cache Size
1	50	59.8	0.0	0.0	267,231	8.9 MB
2	100	56.6	56.5	56.5	472,616	15.7 MB
3	150	38.4	79.3	79.3	596,215	19.8 MB
4	200	82.2	66.4	66.4	897,546	29.9 MB
5	250	108.9	68.6	68.6	1,308,063	43.6 MB
6	300	61.3	88.3	88.3	1,481,540	49.4 MB

Note: Se-1540 dataset with SNPs+InDel-contiguous mode, DNA-strict alignment parameters, and EFSA schema. Each batch contains cumulative samples (batch 2 includes batch 1 samples plus 50 new). Cache efficiency represents the percentage of alignments reused from previous computations. The counter-intuitive performance improvement at batch 6 (300 samples faster than 250 samples) demonstrates the significant impact of high cache hit rates on surveillance scalability.

S9 Recombination-Candidate Flagging — Results

Application of the Flagging Step to Salmonella enterica Se-1540

We applied the recombination-candidate flagging step to the *Salmonella enterica* Se-1540 dataset using a cache enriched with sequence-length information. The resulting output is presented as a shortlist of candidate loci for follow-up investigation and is intended to complement, rather than replace, dedicated recombination-detection tools.

Empirical ρ Distribution and Threshold Selection: Distribution analysis of ρ values in the Se-1540 dataset was used to inform selection of the flagging threshold. The empirical ρ values exhibited a bimodal pattern (modes at 4.9% and 16.5%) with a median of 3.50% and a first quartile of 2.90%. We selected 3.0% as the flagging threshold based on its position near the center of the empirical distribution. We emphasize that this threshold represents a dataset-specific heuristic choice rather than a validated universal cutoff. We do not claim that the 3.0% value generalizes across datasets, species, or cgMLST schemas, nor do we infer sensitivity or specificity in the absence of an independent ground truth validation.

The analysis applied epidemiological filtering (Hamming distance threshold $\leq$15) together with a 3.0% ρ threshold to flag candidate regions across 3,253 EFSA-filtered cgMLST loci. Quality filtering retained samples with $\geq$98% cgMLST completeness.

Applying these filters flagged 20 sample pairs in the Se-1540 dataset, affecting 0.03% of EFSA-filtered loci. Among samples involved in flagged pairs for which metadata were available, all were patient-derived isolates. Most lacked temporal metadata, whereas approximately 50% of the full dataset included collection years spanning 1987–2021. The ρ values observed at flagged loci ranged from the selected threshold (3.0%) to substantially higher values, indicating within-allele divergence well above the background distribution observed across the dataset. Such elevated divergence may reflect horizontal gene transfer, but may also arise from other evolutionary or methodological factors, as discussed above.

Note on Flagged Sample Pairs vs Tier 1-2 Cluster Boundaries

We observed empirically that the 20 flagged sample pairs in Se-1540 lay primarily outside the cluster boundaries identified by the Tier 1-2 distance-based analyses. We report this as a consistency observation rather than as cross-validation between independent analyses, since both derive from the same underlying alignment statistics and would therefore be expected to show partial agreement by construction. A formal assessment of whether the flagged regions correspond to genuine horizontal gene transfer events would require comparison against established recombination-detection tools (e.g., Gubbins [1], ClonalFrameML [2], fastGEAR [3]) and is beyond the scope of the present study.

The flagging step described above is presented as a preliminary feasibility demonstration, illustrating how the cgDist cache architecture can support an exploratory companion analysis without additional computational overhead. Comprehensive evaluation of the analytical performance and epidemiological utility of this flagging approach, including direct benchmarking against the established tools cited above, is reserved for future work.

References

[1] Nicholas J. Croucher, Andrew J. Page, Thomas R. Connor, Aidan J. Delaney, Jacqueline A. Keane, Stephen D. Bentley, Julian Parkhill, and Simon R. Harris. Rapid phylogenetic analysis of large samples of recombinant bacterial whole genome sequences using gubbins. *Nucleic Acids Research*, 43(3):e15, 2015. doi: 10.1093/nar/gku1196.

[2] Xavier Didelot and Daniel J. Wilson. ClonalFrameML: efficient inference of recombination in whole bacterial genomes. *PLoS Computational Biology*, 11(2):e1004041, 2015. doi: 10.1371/journal.pcbi.1004041.

[3] Rafal Mostowy, Nicholas J. Croucher, Cheryl P. Andam, Jukka Corander, William P. Hanage, and Pekka Marttinen. Efficient inference of recent and ancestral recombination within bacterial populations. *Molecular Biology and Evolution*, 34(5):1167–1182, 2017. doi: 10.1093/molbev/msx066.

[4] Torsten Seemann. cgmlst-dists: Calculate distance matrix from chewbbaca cgmlst allele call tables. `https://github.com/tseemann/cgmlst-dists`, 2020. Accessed: 2025-04-23.
